# Polarization of Rheumatoid Macrophages by TNF Targeting Through an IL-10/STAT3 Mechanism

**DOI:** 10.3389/fimmu.2019.00003

**Published:** 2019-01-18

**Authors:** Yannick Degboé, Benjamin Rauwel, Michel Baron, Jean-Frédéric Boyer, Adeline Ruyssen-Witrand, Arnaud Constantin, Jean-Luc Davignon

**Affiliations:** ^1^Centre de Physiopathologie Toulouse Purpan, INSERM UMR 1043, Toulouse, France; ^2^Centre de Rhumatologie, CHU de Toulouse, Toulouse, France; ^3^Faculté de Médecine, Université Paul Sabatier Toulouse III, Toulouse, France; ^4^UMR1027, INSERM—Université Paul Sabatier Toulouse III, Toulouse, France

**Keywords:** macrophage, alternative polarization, anti-TNF agents, TNF, interleukin 10, STAT3

## Abstract

Macrophages contribute to the pathogenesis of rheumatoid arthritis (RA). They can display different states of activation or “polarization,” notably the so-called inflammatory “M1” and the various alternative “M2” polarizations, characterized by distinct functions. Data regarding the effects of RA anti-cytokine biological disease-modifying anti-rheumatic drugs (bDMARDs) on macrophage polarization are scarce. We aimed to assess *in vitro* modulation of macrophage polarization by bDMARDs targeting pro-inflammatory cytokines in RA. We generated monocyte derived macrophages using blood samples from 20 RA patients with active RA and 30 healthy controls. We evaluated *in vitro* the impact on M1 inflammatory macrophages of: etanercept (ETA), adalimumab (ADA), certolizumab (CZP), tocilizumab (TCZ), and rituximab (RTX). We assessed the impact on macrophage polarization using flow cytometry and RTqPCR to study the expression of surface markers and perform functional studies of cytokine production, phagocytosis, and negative feedback control of inflammation. Among evaluated bDMARDs, anti-TNF agents modulated the polarization of inflammatory macrophages by decreasing inflammatory surface markers (CD40, CD80) and favoring alternative markers (CD16, CD163, MerTK). Anti-TNF agents also induced alternative functions in macrophages activated in inflammatory condition with (i) the inhibition of inflammatory cytokines (TNF, IL-6, IL-12), (ii) an increase in phagocytosis. These findings were mechanistically related to an increase in early IL-10 production, responsible for higher negative feedback control of inflammation involving SOCS3 and Gas6. This IL-10 effect was STAT3-dependent. Anti-TNF agents not only inhibit *in vitro* inflammatory functions of macrophages, but also favor resolution of inflammation through polarization toward alternative features specifically involving the IL-10/STAT3 axis.

## Introduction

Plasticity is a key feature of macrophages. They can be activated by many stimuli, depending on their environment, resulting in specific states of polarization. Their plasticity is illustrated by various profiles including the so-called M1 pro-inflammatory polarization, and M2 alternative polarizations driving immunoregulatory and wound-healing functions (1, 2). The M1/M2 historical classification is far too simplistic to describe the variety of macrophage phenotypes (3), especially with regards to rheumatoid arthritis (RA).

Although a few studies on synovial tissue failed to identify specific macrophage polarization surface markers in RA synovium (4, 5), several studies have clearly demonstrated that infiltrating synovial macrophages display a pro-inflammatory profile (6). Macrophages play a central effector role in rheumatoid synovitis. They contribute to the inflammatory environment: by producing pro-inflammatory cytokines, especially TNF (7), by enhancing synovial homing of immune cells through chemokines (CCL2, CCL3, CCL5, IL-8, CX3CL1) (8), by producing angiogenic factors (Vascular Endothelial Growth Factor, Fibroblast Growth Factor-β, Platelet-Derived Growth Factor) (9–12), and by inducing oxidative damage through reactive oxygen species (13). Macrophages crosstalk with CD4 T cells is able to induce Th1 and Th17 commitment as identified in RA (14, 15). Moreover, they activate fibroblastic synoviocytes and induce osteoclastogenesis thus contributing to joint destruction in RA (16, 17). In addition to their well-described role in the effector phase of RA, macrophages may also contribute to the disease onset, especially by generating arthritogenic citrullinated peptides (18, 19).

Synovitis in active RA is fueled by macrophages generated from newly differentiated blood monocytes. In therapeutic context, those monocytes-derived macrophages are concomitantly exposed to differentiating and activating stimuli as well as to drugs. Infiltration of the synovial sublining by CD68+ macrophages is a biomarker of disease severity and therapeutic response (20). However, data concerning the effects of RA biological disease-modifying anti-rheumatic drugs (bDMARDs) on macrophage polarization are scarce. Anti-cytokine bDMARDs have been shown to reduce the inflammatory burden provided especially by recruited inflammatory monocytes/macrophages (21–23). Whether or not an alternative polarization of macrophages is related to the therapeutic response in RA remains unknown. This issue is a key point to better understand the mechanism of action of bDMARDs in RA, and to tailor the therapeutic targeting of macrophages in RA.

In the current study, we aimed to assess *in vitro* modulation of monocyte-derived macrophage polarization of RA patients by bDMARDs, especially anti-TNF agents. We found that anti-TNF polarize macrophage toward an alternative pro-resolving phenotype.

## Methods

### Study Participants

Peripheral blood mononuclear cells (PBMC) were obtained from 20 RA patients and 30 healthy controls. RA patients were recruited in the Rheumatology Center of the Toulouse University Hospital (CHU Toulouse, France). Inclusion criteria were: age ≥18 years old, RA diagnosis according to the ACR/EULAR 2010 criteria, active RA (DAS28 ≥2.6) and indication for a first or second bDMARD initiation. Blood samples of RA patients were collected before initiation of the bDMARD. Due to the potential effects of corticosteroids on macrophage polarization, we excluded steroid (GC) use >10 mg prednisone equivalent/day, IV use of steroids, or intra-articular injection of steroids < 2 weeks before. Healthy controls were recruited from the Etablissement Français du Sang (Toulouse, France). Informed written consent was obtained, and the study protocol regarding RA patients was approved by the local ethics committee (CHU Toulouse—BioTOUL DC 2016–2804).

### Generation of Macrophages

CD14+ monocytes were purified by positive magnetic sorting (Affymetrix), from PBMC isolated on Pancoll (Pan Biotech). Purity was measured by flow cytometry (MACSQuant 10, Miltenyi), using a CD14-FITC antibody (clone HCD14, BioLegend). Sample purity was routinely ≥95%. Macrophages were derived from monocytes (MDM). Monocytes (0.5 × 10^6^/ml) were differentiated into macrophages in the presence of recombinant M-CSF (50 ng/ml; BioLegend) for 5 days. Cells were cultivated at 37°C/5% CO_2_ in RPMI medium 1640 + Glutamax (Gibco), supplemented with 10% fetal calf serum (Gibco), Penicillin G (Gibco), and Streptomycin (Gibco).

### Activation of Macrophages

MDM were activated or not for 24 h as either M1 pro-inflammatory MDM using LPS from *E. coli* (20 ng/ml; Sigma-Aldrich) and IFNγ (25 ng/ml; Peprotech), or M(IL10) alternative MDM using IL-10 (50 ng/ml; Peprotech), or M(IL4) alternative MDM using IL-4 (25 ng/ml; Peprotech) (24).

M1 MDM were cultivated with or without bDMARDs, during the 24 h activation phase. The bDMARDs were used at 10 μg/ml. We evaluated 2 anti-TNF agents [etanercept (ETA), adalimumab (ADA)], 1 anti-IL6-receptor agent [tocilizumab (TCZ)], and 1 anti-CD20 agent [rituximab (RTX); as a control of unspecific impact of the Fc fragment]. Certolizumab (CZP) was used in some experiments.

### Flow Cytometry Analysis

We assessed the effects of bDMARDs on M1 activation by a flow cytometric analysis of membrane markers. Before labeling, MDM were blocked with a Fc receptor blocking solution: Human TruStain FcX (BioLegend). Surface staining was performed using the following murine anti-human antibodies: CD40 APC/Cy7 (clone 5C3, BioLegend), CD80 BV421 (clone 2D10, BioLegend), CD206 AF488 (clone 15-2, BioLegend), CD200R PE (clone OX-108, BioLegend), CD64 PC7 (clone 10.1, BioLegend), MER proto-oncogene tyrosine kinase (MerTK) PE (clone 125518, R&D systems), CD163 FITC (clone GHI/61.1, Miltenyi), CD16 V500 (clone 3G8, BD Biosciences). We evaluated median fluorescence intensity (MFI). Given the high auto-fluorescence of the macrophages, and the variability of this auto-fluorescence depending on the stimulation, fluorescence levels were expressed as ratio (specific labeling/corresponding isotype).

For intra-cellular staining of phospho-STAT3, cells were fixed and permeabilized with a Transcription Factor Buffer Set (BD), following the manufacturer's protocol. We performed a primary labeling with a rabbit anti-human phospho-Stat3 (Tyr705) (clone D3A7, Cell Signaling Technology) and a secondary labeling with an anti-Rabbit IgG (H+L), F(ab')_2_Fragment (Alexa Fluor 647 Conjugate; Cell Signaling Technology).

Cells were analyzed on a MACSQuant 10 (Miltenyi). Data were analyzed using FlowJo v7.6.5 (Tree Star).

### Cytokine Measurements

Culture supernatants were collected and stored at −80°C until analysis. Concentrations of IL-6, IL-10, IL-12, and TNF were determined simultaneously using Cytometric Bead Array (Human Flex set, BD Biosciences). Data acquisition was performed on a LSRII (BD Biosciences) and analysis was performed using FCAP Array v3 (Soft Flow). TGFβ was quantitated by ELISA (Ready-SET-Go, eBioscience, San Diego, CA, USA) on a Varioskan Flash (Thermo Scientific) spectrophotometer and analyzed using the SkanIt™ (Thermo Scientific) program.

### Gene Expression Analysis

Total RNA from 1.5 × 10^6^ monocytes was isolated using High Pure RNA Isolation Kit (Roche Diagnostics GmbH, Mannheim, Germany) and complementary DNA (cDNA) synthesized with RevertAid Minus Reverse Transcriptase (Thermo Fisher Scientific, Waltham, MA, USA). Gene expression was performed using LightCycler 480 SYBR Green Master Mix (Roche Diagnostics GmbH) and a LightCycler 480 System instrument (Roche Diagnostics GmbH). All primers were designed using ProbeFinder Software (Roche Applied Science website), and synthesized by Sigma Life Science (St Quentin Fallavier, France).

GAPDH forward: 5′-acccactcctccacctttgac-3′, GAPDH reverse: 5′ -ctgttgctgtagccaaattcgt-3′

TNFα forward: 5′-cagcctcttctccttcctga-3′, TNFα reverse: 5′-acccactcctccacctttgac-3′

IL10 forward: 5′-aacaagagcaaggccgtgg−3′, IL10 reverse: 5′-gaagatgtcaaactcactcatggc-3′

GAS6 forward: 5′- acctcatgggcaacttcttc-3′, GAS6 reverse: 5′-ggctgcattcgttgacatc-3′

SOCS3 forward: 5′-agacttcgattcgggacca-3′, SOCS3 reverse: 5′-aacttgctgtgggtgaccat-3′

### Phagocytosis Assay

Phagocytosis ability was assessed by flow cytometry using pHrodo green *E.coli* bioparticles®, following the protocol recommended by the manufacturer. Briefly, 100,000 cells (1,000,000/mL) per well were cultivated in a 96-well plate. They were activated for 24 h as M1 in the presence or absence of bDMARD. Then, culture medium was replaced by 100 μL of pHrodo bioparticles resuspended in Dulbecco's phosphate-buffered saline (DPBS, Gibco), and incubated for 90 min at 37°C/5% CO_2_. Cells were then harvested after 10 min of incubation on ice in cold DPBS and assessed by flow cytometry (MACSQuant Q10). In some experiments, inhibition of CD16-dependent phagocytosis was performed during the activation phase, with a LEAF purified anti-human CD16 antibody (clone 3G8, BioLegend) used at 10 μg/ml. All conditions were performed in duplicate.

### Western Blot Analysis

Total extracts from 2 × 10^6^ monocytes lysed in 50 μl of Laemmli buffer were denatured at 95°C for 10 min and sonicated: 15–20 μl were run on Novex NuPAGE 4–12 % Bis-Tris mini gels and transferred on nitrocellulose membrane with X-Cell blot module (Life Technologies). After incubation with primary (phospho-STAT3 Tyr 705—clone D3A7—Cell Signaling Technology; STAT3—clone D1A5 - Cell Signaling Technology) and secondary HRP-coupled antibodies, labeled proteins were visualized by enhanced chemiluminescence with ECL Prime Western Blotting Detection Reagent (GE Healthcare, Piscataway, NJ, USA) by a ChemiDoc XRS+ imaging system (Bio-Rad Laboratories, Hercules, CA, USA). All images were analyzed with the Image Lab 5.0 software (Bio-Rad).

### Pharmacological Inhibition of STAT3

MDM activated for 24 h as M1 MDM were cultivated during this activation phase with cucurbitacin I (50 mM, Sigma) a selective JAK2/STAT3 inhibitor, or with STATTIC (1 μM, Selleckchem) an inhibitor of phospho-tyrosine binding to the SH2 domain of STAT3.

### Statistical Analysis

In most of the experiments, non-activated MDM and M1 MDM cultivated in the presence of bDMARDs were compared to untreated M1 MDM by Sigma Plot v12.5 (Systat Software Inc.). We tested whether values had a gaussian distribution (Shapiro-Wilk test). In case of Gaussian distribution, samples were compared using a Student paired *t*-test; otherwise, medians were compared by a Wilcoxon matched pairs test. Multiple groups comparison was performed by a One-way ANOVA and Bonferroni's post-test, or Kruskal-Wallis test and Dunn's post-test in case of non-Gaussian distribution. We evaluated the correlation between CD16 membrane expression in MDM and DAS28 ESR at inclusion by the Pearson r correlation coefficient. We evaluated the association between CD16 expression in MDM (categorized as low i.e., ≤ 2.1 vs. high i.e., >2.1) and DAS28 ESR at inclusion (categorized as low-to-moderate i.e., ≤ 5.1 vs. high i.e., >5.1) by a Fisher's exact test and evaluated the strength of this association with odds ratio (OR) calculation. We assessed the influence of the background treatment regimen [GC, methotrexate (MTX)] on the expression of polarization markers in the different conditions of activation, by a two-way ANOVA. A *p* value < 0.05 was considered to be significant, with: ^***^p value < 0.001, ^**^0.001 ≤ *p* < 0.1, ^*^0.01 ≤ *p* < 0.05.

## Results

### Patient Characteristics

We included 20 RA patients with active RA and 30 healthy controls. Blood samples were collected in 15/20 patients (75%) before initiation of the first bDMARD, and in 5/20 patients (25%) before initiation of the second bDMARD. Choice of bDMARD therapy was determined by the treating physician: etanercept in 9 patients, adalimumab in 3 patients, rituximab in 3 patients, tocilizumab in 2 patients, abatacept in 1 patient, golimumab in 1 patient, and certolizumab in 1 patient. At inclusion, 9 patients (45.0%) had MTX background, and 6 patients (30.0%) had GC background. The patient characteristics are provided in Table 1.

**Table 1:** Characteristics of patients with rheumatoid arthritis at bDMARD initiation.

	**RA population (N = 20)**
**DEMOGRAPHIC DATA**	
Age (year), mean (SD)	63.4 (13.2)
Women (%)	12 (60)
Disease duration (year), mean (SD)	11.3 (9.7)
**CLINICAL DATA**	
DAS28 ESR, mean (SD)	4.63 (1.32)
Erosive RA (%)	14 (73.7)
Steroid use (%)	6 (30.0)
Daily steroid dose (mg prednisone/day), median (IQR)	7.5 (5.0 – 10.0)
csDMARDs, MTX (%)	9 (45.0)
**BIOLOGICAL DATA**	
ESR (mm/h), median (IQR)	16.0 (12.0 – 25.0)
CRP (mg/L), median (IQR)	6.7 (1.8 – 15.5)
RF+ (%)	18 (94.7)
Anti-CCP+ (%)	18 (94.7)
Leucocytes/mm^3^, mean (SD)	8252 (3058)
Monocytes/mm^3^, mean (SD)	578 (315)

*bDMARD, biological disease-modifying anti-rheumatic drugs; csDMARDs, conventional synthetic disease-modifying anti-rheumatic drugs; CCP, cyclic citrullinated peptides; CRP, C reactive protein; DAS28 ESR, disease activity score 28 ESR; ESR, erythrocyte sedimentation rate; IQR, interquartile range; MTX, methotrexate; RA, rheumatoid arthritis; RF, rheumatoid factor; SD, standard deviation*.

### Validation of Surface Markers of Macrophage Polarization

**Figure 1:**
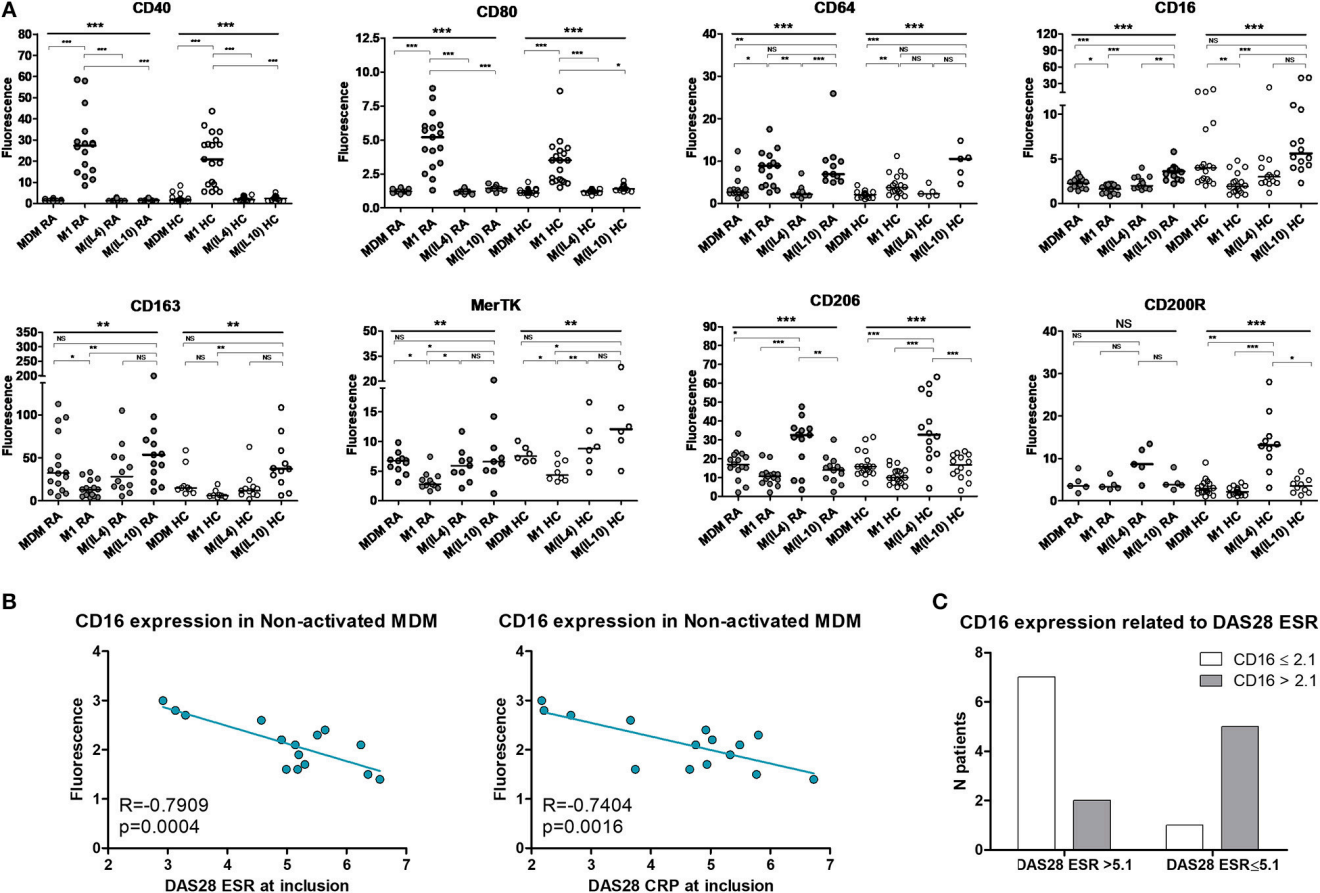
Surface polarization markers on macrophages. MDM, monocyte-derived macrophages; M1, pro-inflammatory macrophages activated by LPS + IFNγ; M(IL4), alternative macrophages activated by IL-4; M(IL10), alternative macrophages activated by IL-10; RA, rheumatoid arthritis; HC, healthy controls; DAS28, disease activity score 28; ESR, Erythrocyte sedimentation rate; CRP, C reactive protein; NS, non-significant. **(A)** Surface markers on macrophages from up to 16 RA patients (gray circles) and 20 healthy controls (open circles) were analyzed by flow cytometry, after differentiation (MDM) and subsequent 24 h activation as M1, or M(IL4), or M(IL10). **(B)** Correlation (Pearson) between CD16 membrane expression in MDM and RA activity assessed by the DAS28 ESR and DAS28 CRP scores. **(C)** CD16 membrane expression in MDM from patients with active RA, according to disease activity (DAS28 ESR). ^***^p value < 0.001, ^**^0.001 ≤ *p* < 0.01, ^*^0.01 ≤ *p* < 0.05 (One-way ANOVA and Bonferroni's post-test, or Kruskal-Wallis test and Dunn's post-test).

PBMC from 16 RA patients and 20 controls were differentiated into MDM and activated or not as M1 pro-inflammatory macrophages, M(IL4) alternative macrophages, and M(IL10) alternative macrophages (Figure 1A). The possibility of using surface markers to discriminate these different polarization states was assessed by flow cytometry. In RA patients, CD40 and CD80 were highly expressed in M1 macrophages, but poorly expressed in MDM and M2 macrophages. We considered these markers as M1 markers. CD16 and CD163 were highly expressed in M(IL10), expressed at an intermediate level in MDM and M(IL4), and poorly expressed in M1 macrophages. We thus considered CD16 and CD163 as M(IL10) markers. MerTK was expressed at a similar level, i.e., intermediate, in MDM, M(IL4), and M(IL10), and at lower level in M1, leading us to consider MerTK as a pan-M2 marker. CD206 and CD200R were expressed at high level in M(IL4), at low-to-intermediate level in MDM and M(IL10), and at lower level in M1 macrophages, leading us to consider them as M(IL4) markers. CD64 was associated with both M1 and M(IL10) polarization states, thus non-discriminative for further experiments.

When compared to controls, RA patients macrophages displayed a biased plasticity. They were prone to express higher level of inflammatory markers after M1 activation (CD40, CD80, CD64), and lower level of M(IL4) markers (CD206, CD200R) and M(IL10) markers (CD16, CD64) in the *ad hoc* activation condition. Surprisingly, CD163 expression was higher in macrophages from RA patients. Despite these differences, discrimination using selected surface markers was similar in RA patients and in healthy controls.

We next confirmed the functional relevance of those polarization markers in RA patients. Among the selected markers, only CD16 membrane expression in MDM was specifically correlated with disease activity measured at inclusion by the DAS28 ESR and DAS28 CRP (Figure 1B). In our model, CD16 was considered as a marker of alternative polarization [M(IL10)]. We categorized and CD16 expression as high (>2.1) and low (≤ 2.1), and disease activity as high (DAS28 ESR > 5.1) and low-to-moderate (2.6 < DAS28 ESR ≤ 5.1). We observed a significant association between high CD16 expression in MDM and low-to-moderate disease activity at inclusion (Fisher's exact test *p* value = 0.0406, OR = 17.5 [1.2-250.5]; Figure 1C).

### Anti-TNF Agents Favor Alternative Over Pro-Inflammatory Surface Markers

**Figure 2:**
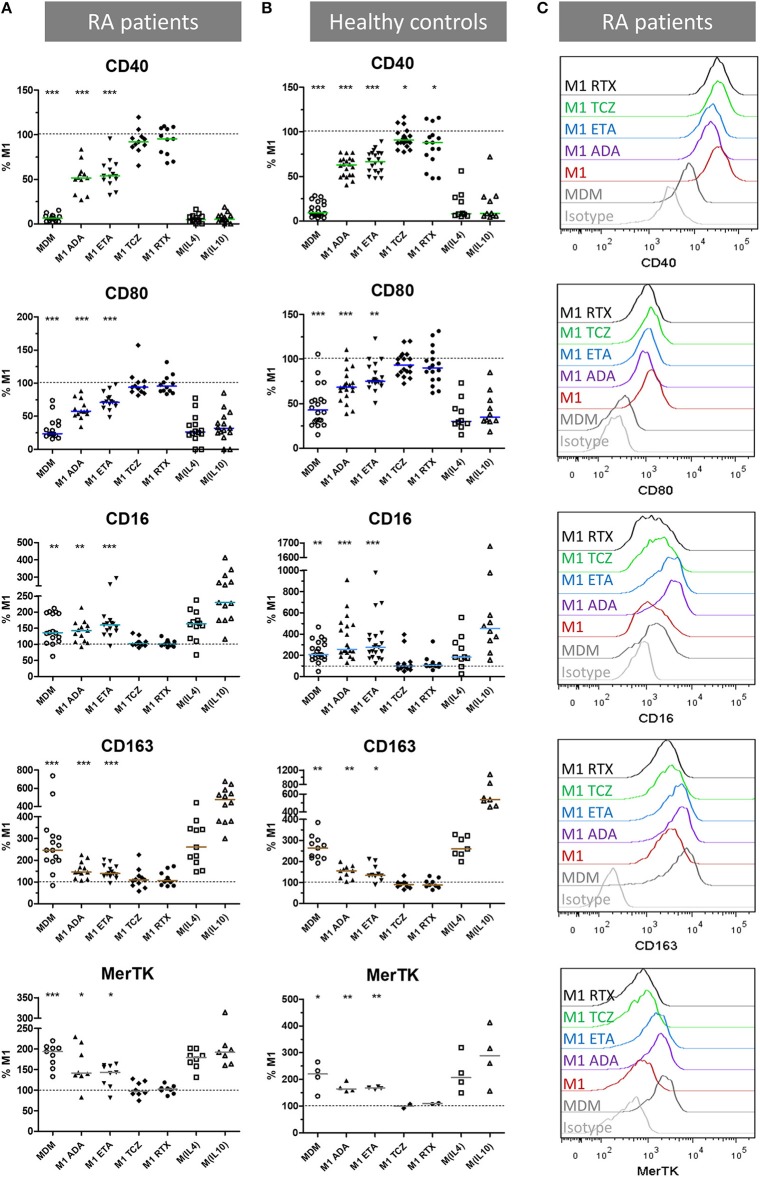
Anti-TNF agents favor alternative polarization of macrophages. MDM, monocyte-derived macrophages; M1, pro-inflammatory macrophages activated by LPS + IFNγ; M(IL4), alternative macrophages activated by IL-4; M(IL10), alternative macrophages activated by IL-10; ADA, adalimumab; ETA, etanercept; TCZ, tocilizumab; RTX, rituximab; RA, rheumatoid arthritis. **(A–C)** Monocyte-derived macrophages were activated for 24 h as M1, M(IL4), and M(IL10) with or without the indicated bDMARD. Surface polarization markers on macrophages from up to 16 RA patients **(A,C)** and 21 healthy controls **(B)** were analyzed by flow cytometry. Results are standardized to the level of surface markers expressed in M1 untreated condition. Non-activated MDM and M1 MDM activated in the presence of the indicated bDMARD were compared to M1 MDM. ^***^p value < 0.001, ^**^0.001 ≤ *p* < 0.01, ^*^0.01 ≤ *p* < 0.05 (Student paired *t*-test or Wilcoxon matched pairs test).

We then assessed the impact of anti-TNF agents (ADA, ETA), the anti-IL-6 receptor drug (TCZ), and RTX used as control on polarization surface markers in MDM from 16 RA patients and 21 healthy controls activated as M1 pro-inflammatory macrophages (Figure 2, Supplemental Figures S1–S2). In RA patients as well as in controls, compared to M1 untreated macrophages, anti-TNF agents induced a significant modulation of polarization surface markers: (i) a decrease in M1 markers (CD40 and CD80), (ii) an increase in M(IL10) markers (CD16, CD163) and in the pan M2 marker MerTK (Figure 2, Supplemental Figure S1), and (iii) no effect on M(IL4) markers (CD206 and CD200R) (Supplemental Figure S2). Although the modulation of polarization markers by anti-TNF agents was similar in RA patients and controls, decrease in CD40 was observed with a greater extent in RA patients and increase in CD16 was observed with a greater extent in controls.

TCZ induced a slight but significant CD40 decrease in controls, and a slight but significant increase in CD206 in RA patients and controls. The anti-CD20 drug (RTX) slightly but significantly reduced CD40 in controls.

To take into account the possible impact of background treatment regimen at inclusion on our results, we evaluated the influence of prior exposure to GC < 10 mg/day, MTX and bDMARD on the *in vitro* modulation of membrane polarization markers by bDMARDs (Supplemental Figure S3). Prior exposure to GC and MTX slightly impacted the expression of polarization markers. GC background (Supplemental Figure S3A) accounted for: (i) 5.7% of the total variance of CD80 expression (*p* = 0.0053), (ii) and 7.6% of the total variance of MerTK expression (*p* = 0.0146). MTX background (Supplemental Figure S3B) accounted for: (i) 16.0% of the total variance of CD206 expression (*p* < 0.0001), (ii) and 3.4% of the total variance of CD64 expression (*p* = 0.0168). Prior exposure to bDMARD impacted the expression of polarization markers. bDMARD background (Supplemental Figure S3C) accounted for: (i) 6.7% of the total variance of CD163 expression (*p* = 0.0225), (ii) 41.0% of the total variance of MerTK expression (*p* < 0.0001), (iii), and 9.9% of the total variance of CD64 (*p* = 0.0015).

To take into account the contribution of the Fc fragment of the fusion protein or monoclonal antibodies used in our experiments on polarization, we compared ETA and ADA to CZP (Fc-free PEGylated TNF inhibitor fab fragment), and RTX (with Fc but not relevant for macrophages, because it targets CD20). Since we observed similar surface marker patterns with ETA, ADA, and CZP, but not with RTX, we considered the modulation of polarization surface markers by anti-TNF agents to be independent of the presence of Fc (Supplemental Figure S4).

### Anti-TNF Agents Decrease the Production of Pro-Inflammatory Cytokines

We next investigated whether phenotypic changes were associated with functional changes.

**Figure 3:**
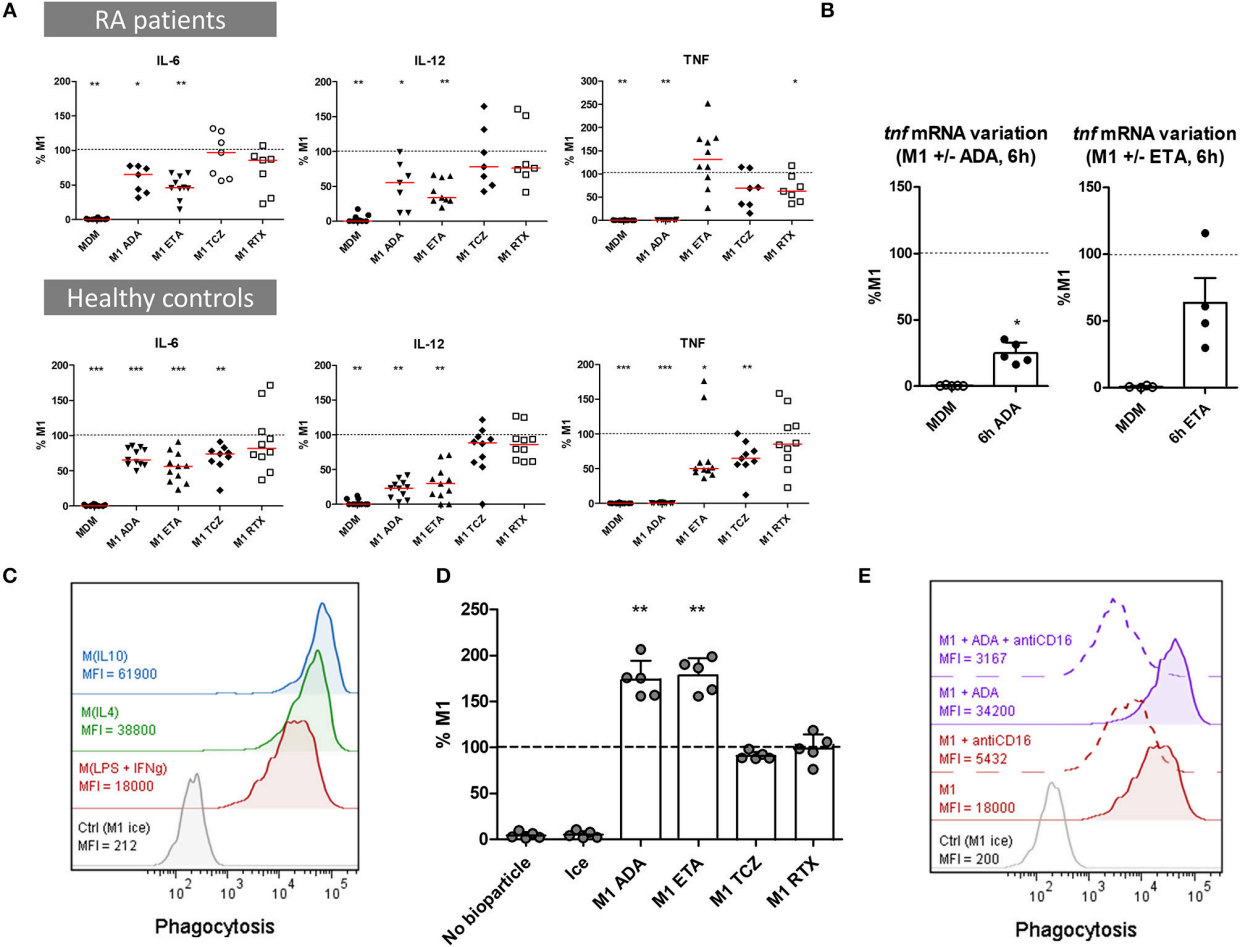
Anti-TNF agents modulate macrophages functions by inhibiting pro-inflammatory cytokine production and enhancing phagocytosis. MDM, monocyte-derived macrophages; M1, pro-inflammatory macrophages activated by LPS + IFNγ; M(IL4), alternative macrophages activated by IL-4; M(IL10), alternative macrophages activated by IL-10; RA, rheumatoid arthritis; ADA, adalimumab; ETA, etanercept; TCZ, tocilizumab; RTX, rituximab; RA, rheumatoid arthritis. Monocytes derived macrophages were activated for 24 h as M1, M(IL4), and M(IL10) with or without the indicated bDMARD. **(A)** Cytokines production was measured in cell culture supernatant after 1 day of activation by cytometric bead array. Results are standardized to cytokine level in M1 untreated condition. **(B)** Tnf mRNA was analyzed in non-activated MDM (Baseline) and in M1 inflammatory macrophages activated 6 h with or without anti-TNF agents. Results are normalized to mRNA level in M1 macrophages (up to 5 healthy controls). **(C–E)** Phagocytosis of green-labeled *E.coli* particles was assessed by flow cytometry. All experiments were performed in duplicate. Macrophages incubated on ice were used as negative control. **(C)** Phagocytosis was assessed in 24 h activated M1, M(IL4), and M(IL10). Data representative for 3 healthy controls. **(D)** The effect of bDMARD on phagocytosis by M1 macrophages was assessed by flow cytometry (5 healthy controls). **(E)** The impact of CD16 blockade on phagocytosis was assessed by flow cytometry. MDM were activated as M1 macrophages for 24 h in the presence or not of ADA and an anti-CD16 blocking antibody (10 μg/ml). Data representative for 4 healthy controls. Non-activated MDM and M1 MDM activated in the presence of bDMARDs were compared to untreated M1 MDM. ^***^p value < 0.001, ^**^0.001 ≤ *p* < 0.01, ^*^0.01 ≤ *p* < 0.05 (Student paired *t*-test or Wilcoxon matched pairs test).

We evaluated the impact of bDMARDs on the secretion of soluble pro-inflammatory cytokines (TNF, IL-6, IL-12) in cell culture supernatants, and on mRNA expression (TNF). Cytokine secretion was assessed in MDM from 10 RA patients and 12 healthy controls activated as M1 pro-inflammatory macrophages (Figure 3A, Supplemental Table S1). In RA patients as well as in controls, anti-TNF agents induced a significant decrease in pro-inflammatory cytokines compared to M1 untreated macrophages. Notably, we observed a strong decrease in IL-12 and IL-6.

ADA competed with antibodies in TNF detection assays and thus prevented the detection of soluble TNF in supernatants (Supplemental Figure S5A). However, *tnf* mRNA decreased from 6 h post-activation in M1 MDM treated with ADA when compared to non-treated M1 MDM, indicating thus an inhibition of TNF gene expression by ADA (Figure 3B).

The concentration of ETA was set to inhibit pro-inflammatory cytokines production and inhibit the pro-inflammatory phenotype. TNF secretion was decreased by ETA from the first hours of M1 activation (Supplemental Figure S6). At 24 h post-activation, we observed a dose-dependent inhibition of TNF concentration by ETA. ETA at 10 μg/mL did not affect TNF secretion in RA patients, and significantly decreased TNF in controls, who notably secreted lower levels of TNF (Supplemental Table S1). However, a high concentration of ETA (100 μg/mL) was associated with a reduced secretion of TNF in cell culture supernatants from controls (Supplemental Figure S5B).

TCZ significantly decreased IL6 and TNF production in healthy controls but not in RA patients.

RTX slightly, but significantly decreased TNF in RA patients.

### Anti-TNF Agents Increase Phagocytic Capacity

Phagocytosis is the hallmark of macrophages. Clearance of debris and apoptotic bodies contributes to resolve of inflammation. We assessed the impact of bDMARDs on the phagocytic capacity of MDM from 5 healthy controls activated as M1 pro-inflammatory MDM. Phagocytosis was higher in M(IL10) and M(IL4) activated MDM than in M1 MDM (Figure 3C). Anti-TNF agents, but neither TCZ nor RTX, induced an increase of phagocytosis in M1 MDM (Figure 3D, Supplemental Figure S7). Considering that CD16 is known to be implicated in phagocytosis, and that surface expression of CD16 is increased in our study by anti-TNF agents, we assessed its contribution to *in vitro* phagocytosis by a specific inhibition with a blocking anti-CD16 antibody. The results showed that this increase in phagocytosis by ADA treatment was, at least in part, CD16-dependent (Figure 3E).

### The Impact of Anti-TNF Agents on Macrophage Polarization Involves IL10

**Figure 4:**
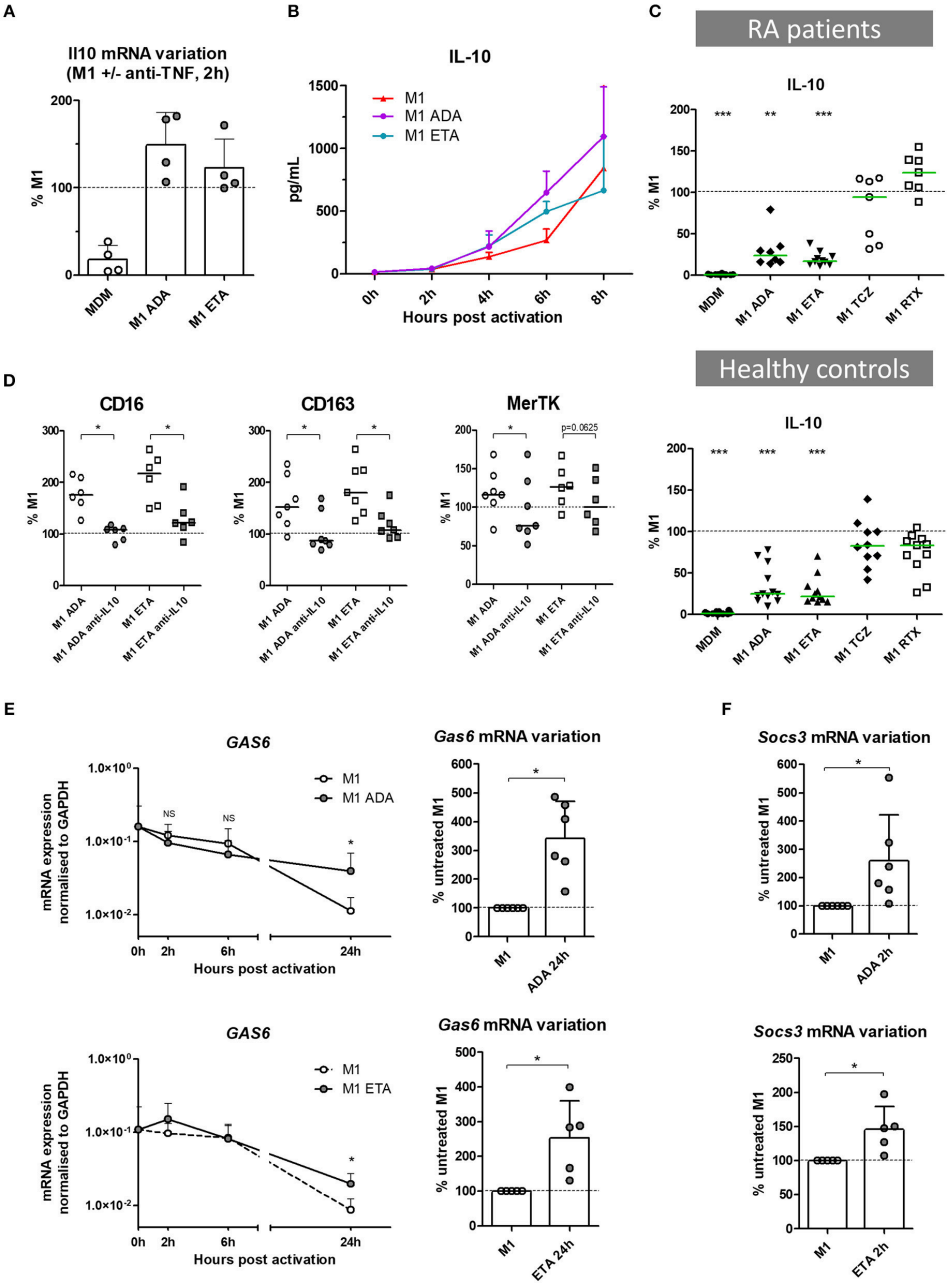
The impact of anti-TNF agents on macrophage polarization involves IL10 negative feedback control of inflammation. MDM, monocyte-derived macrophages; M1, pro-inflammatory macrophages activated by LPS + IFNγ; ADA, adalimumab; ETA, etanercept; Anti-IL10, anti-IL-10 neutralizing antibody. **(A)** Il10 mRNA were analyzed in non-activated MDM and in M1 inflammatory macrophages activated or not in the presence of anti-TNF agents. Results are normalized to mRNA level in M1 macrophages (4 healthy donors). **(B)** Early IL-10 secretion was measured in cell culture supernatants of treated and untreated M1 macrophages by cytometric bead array (3 healthy donors). **(C)** IL-10 secretion was measured in cell culture supernatants, after 1 day of M1 activation in the presence of the indicated bDMARD, by cytometric bead array (10 RA patients and 11 healthy donors). Results are standardized to IL-10 level in M1 condition. **(D)** M1 macrophages were cultivated 24 h in the presence or not of the indicated anti-TNF and neutralizing anti-IL10 monoclonal antibody (10 μg/mL). The impact on M2 polarization markers modulated by anti-TNF agents was assessed by flow cytometry. **(E)** Expression of gas6 mRNA over time, normalized to gapdh expression, and its variation at 24 h time point, in M1 macrophages activated or not in the presence of anti-TNF agents (up to 6 healthy controls). **(F)** Variation of socs3 mRNA at 2 h time point, in M1 macrophages activated or not in the presence of anti-TNF agents (up to 6 healthy controls). Basal MDM and M1 MDM cultivated in the presence of bDMARDs were compared to M1 MDM. ^***^p value < 0.001, ^**^0.001 ≤ *p* < 0.01, ^*^0.01 ≤ *p* < 0.05 (Student paired *t*-test or Wilcoxon matched pairs test).

In the presence of anti-TNF agents, we observed a selective preservation of M(IL10) markers (but not M(IL4) markers), associated with functional modifications including a lower production of inflammatory cytokines and a higher phagocytic capacity. IL-10 is a major cytokine of the negative feedback control of inflammation and increases phagocytosis. We thus assessed the implication of IL-10 in the modulation of macrophage polarization induced by anti-TNF agents in an M1 inflammatory context (Figures 4A–D, Supplemental Figures S8A–C). *Il10* mRNA was induced by M1 activation with fast kinetics. We observed a biphasic modulation of IL-10 in the presence of anti-TNF agents in M1 MDM. First, at an early time-point *il10* mRNA was increased in the presence of anti-TNF agents (2 h post-activation) (Figure 4A). This finding was consistent with the higher IL-10 secretion in cell culture supernatants, in the presence of anti-TNF agents, notably between 2 and 6 h post-activation (Figure 4B). Second, at 24 h of M1 activation, we observed a decrease in IL-10 with anti-TNF agents but not with TCZ and RTX (Figure 4C, Supplemental Figure S8A). This decrease was potentially related to the neutralization of the inflammatory environment by anti-TNF agents. This biphasic modulation suggested a critical role for early IL-10 production in macrophage polarization in the presence of a TNF blockade. Therefore, we next assessed the impact of the early neutralization of IL-10 on the anti-TNF agents-induced polarization (Supplemental Figure S8B). IL-10 inhibition by a monoclonal antibody (mAb) from the start of the 24 h activation phase resulted in a dramatic decrease in M2 alternative surface markers and an increase in M1 inflammatory surface markers (Figure 4D, Supplemental Figure S8C).

### Anti-TNF Agents Amplify IL-10-Related Negative Control of Inflammation

IL-10 controls the inflammatory process through induction of various proteins including Suppressors of cytokine signaling (SOCS), MerTK and its ligand Gas6 (25, 26). SOCS are a family of intracellular cytokine-inducible proteins known to be major regulators of macrophage phenotypes (27). SOCS3 can act downstream from MerTK, which plays a major role in phagocytosis of apoptotic cells and controls many other functions such as immunoregulation. Notably, MerTK inhibits the Toll-like receptors-mediated innate immune response *via* its ligand Gas6, and *via* an increase in SOCS3 level (28, 29). Using RTqPCR, we aimed to assess the contribution of the negative feedback control of inflammation by Gas6 and SOCS3, on the modulation of macrophage polarization induced by anti-TNF agents. *Gas6* was expressed at baseline. M1 inflammatory activation decreased *gas6* expression. Anti-TNF agents maintained the level of *gas6* expression over time until 24 h post-activation (Figure 4E), with a higher level in the presence of ADA than ETA. M1 activation induced *socs3* expression. Anti-TNF agents enhanced this expression in the first hours post-activation (Figure 4F).

### Anti-TNF Agent-Induced Switch From Inflammatory to Alternative Macrophage Polarization Involves STAT3

**Figure 5:**
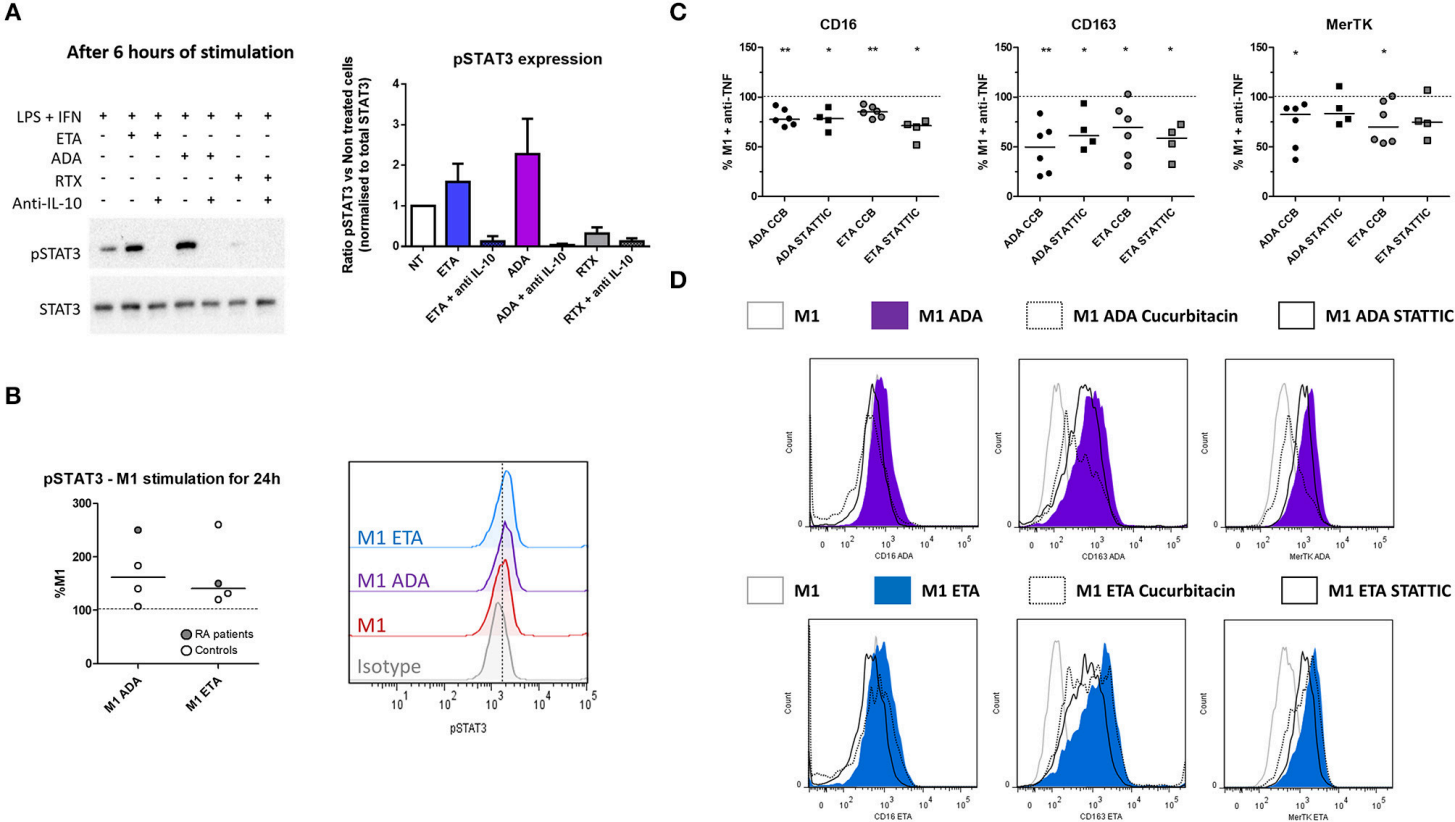
Anti-TNF agent-driven polarization involves an IL-10-dependent induction of STAT3. M1, pro-inflammatory macrophages activated by LPS + IFNγ; ADA, adalimumab; ETA, etanercept; pSTAT3, phospho(Tyr705)-STAT3; RA, rheumatoid arthritis; RTX, rituximab. **(A)** M1 macrophages were cultivated 6 h in the presence or not of the indicated bDMARD and neutralizing anti-IL10 monoclonal antibody (10 μg/mL). The impact of IL-10 neutralization on STAT3 and pSTAT3 expressions were analyzed by western blot. Data representative of 3 independent experiments (representative western blot and quantitative analysis). **(B)** Intracellular staining of pSTAT3 was performed after 24 h of M1 activation with or without anti-TNF agent and analyzed by flow cytometry. **(C,D)** STAT3 inhibitors (Cucurbitacin (circles), CCB 50 nM, STATTIC (squares) 1 μM) were added to the culture medium during the 24 h M1 activation in the presence of the indicated anti-TNF. The subsequent modulation M2 polarization markers induced by anti-TNF agents were assessed by flow cytometry. Non-activated MDM and M1 MDM cultivated in the presence of bDMARDs were compared to M1 MDM. ^**^0.001 ≤ *p* < 0.01, ^*^0.01 ≤ *p* < 0.05 (Student paired *t*-test or Wilcoxon matched pairs test).

Given that IL-10 is involved in the impact of anti-TNF agents on polarization, and that STAT3 is a key transcription factor in M(IL10) commitment, we evaluated the implication of STAT3 in the modulation of macrophage polarization induced by anti-TNF agents in an M1 inflammatory context. We tested whether the early inhibition of IL-10 (mAb) may decrease phospho-STAT3 (pSTAT3) involvement in inflammatory macrophages. We observed an increase in pSTAT3 in the presence of ADA and ETA, and an abolition of pSTAT3 in the presence of the IL-10 mAb (Figure 5A).

In addition to its implication in IL10 signaling, STAT3 is also a key transcription factor in IL-6 signaling. In the presence of LPS, STAT3 activation is transient in the presence of IL-6, whereas it is sustained with IL-10 (30). We observed after 24 h of M1 activation that intracellular expression of pSTAT3 was higher in the presence of anti-TNF than in M1 untreated cells (Figure 5B). Both anti-TNF agents displayed a decreased induction of M(IL10) markers in the presence of selective inhibitors of STAT3: cucurbitacin I and STATTIC (Figures 5C,D, Supplemental Figure S9).

## Discussion

This study demonstrates that anti-TNF agents downregulate surface markers and cytokines associated with an inflammatory phenotype in macrophages, and favor properties such as phagocytosis and negative feedback of inflammation supporting the resolution of inflammation through an IL-10/STAT3 pathway.

Studies addressing the impact of pro-inflammatory cytokine blockades on monocyte/macrophage phenotypes and functions are scarce (21, 31–33). Herein, we provide new data based on macrophages from RA patients that provide new concept of the mode of action of bDMARDs. This is of help to understand the action of anti-TNF bDMARDs in RA.

Infiltrating synovial macrophages originate from blood monocytes. These macrophages are considered as short lifespan cells constantly recruited in the inflammatory synovium: they experience activation-induced cell death (34, 35), apoptosis induced by therapeutic agents (22, 32, 36), and eventually efflux from synovium (37). We used monocyte-derived macrophages submitted to pro-inflammatory conditioning to model the contribution of these infiltrating macrophages to rheumatoid synovitis. In therapeutic condition, newly generated infiltrating macrophages are exposed to the drug from their maturation to their death. Considering the constant turnover of non-resident inflammatory macrophages, it appears that blocking the polarization of macrophages toward an inflammatory phenotype in the presence of anti-TNF is relevant.

Here we have provided data concerning the plasticity of macrophages and discriminative markers of polarization. Some authors opposed M-CSF and granulocyte macrophage colony stimulating factor (GM-CSF) as, respectively, M2 and M1 stimuli. However, this concept is debated (2, 24, 38, 39). Notably, M-CSF may contribute to the inflammatory milieu, with an involvement in collagen induced arthritis (40) and with the induction in macrophages of a high pro-inflammatory response to rheumatoid arthritis-specific immune complex containing ACPA (41). Interestingly, drugs targeting M-CSF are able to inhibit arthritis in murine models of RA (42, 43). As widely proposed (2), we used M-CSF which is known to be the main growth factor involved in macrophages differentiation in homeostasis. As expected, the differentiation process with M-CSF induced a partial M2 “alternative” phenotype bias in MDM that was reversible with pro-inflammatory activation provided by TLR4 ligand + IFNγ.

Anti-TNF agents are commonly used in RA patients. In the current study, we took ADA as a representative of anti-TNF monoclonal antibodies, ETA as a soluble anti-TNF receptor, and CZP as an anti-TNF agent devoid of the Fc fragment. Anti-TNF agents were used at 10 μg/ml, a concentration corresponding to the steady-state level in clinical studies.

Our current study demonstrated that in inflammatory conditions, anti-TNF agents but not TCZ favor CD16, CD163, and MerTK expression while decreasing CD40 and CD80. Ambarus et al. (44) depicted the impact of polarizing cytokines on macrophage surface markers of monocyte-derived macrophages from healthy controls. Even though the experimental protocols were different, our results for polarization markers were consistent with their study. Moreover, we have discovered suitable surface markers of macrophage polarization in RA patients. The discriminative ability of our selected polarization surface markers was similar in RA patients and in controls. To our knowledge, this work is the first to provide such data for RA patients.

A functional hallmark of macrophages is the production of cytokines. As expected from treatments neutralizing a major inflammatory cytokine, anti-TNF agents decreased the production of inflammatory cytokines (IL-6, IL-12, TNF). This decrease is probably due to a blockade of the inflammatory positive loop. In most of the experiments, anti-TNF agents displayed similar properties. Only high concentrations of ETA were able to drastically inhibit TNF production in cell culture supernatants, suggesting a positive loop of TNF regulation that is lost only when TNF is completely neutralized.

**Figure 6:**
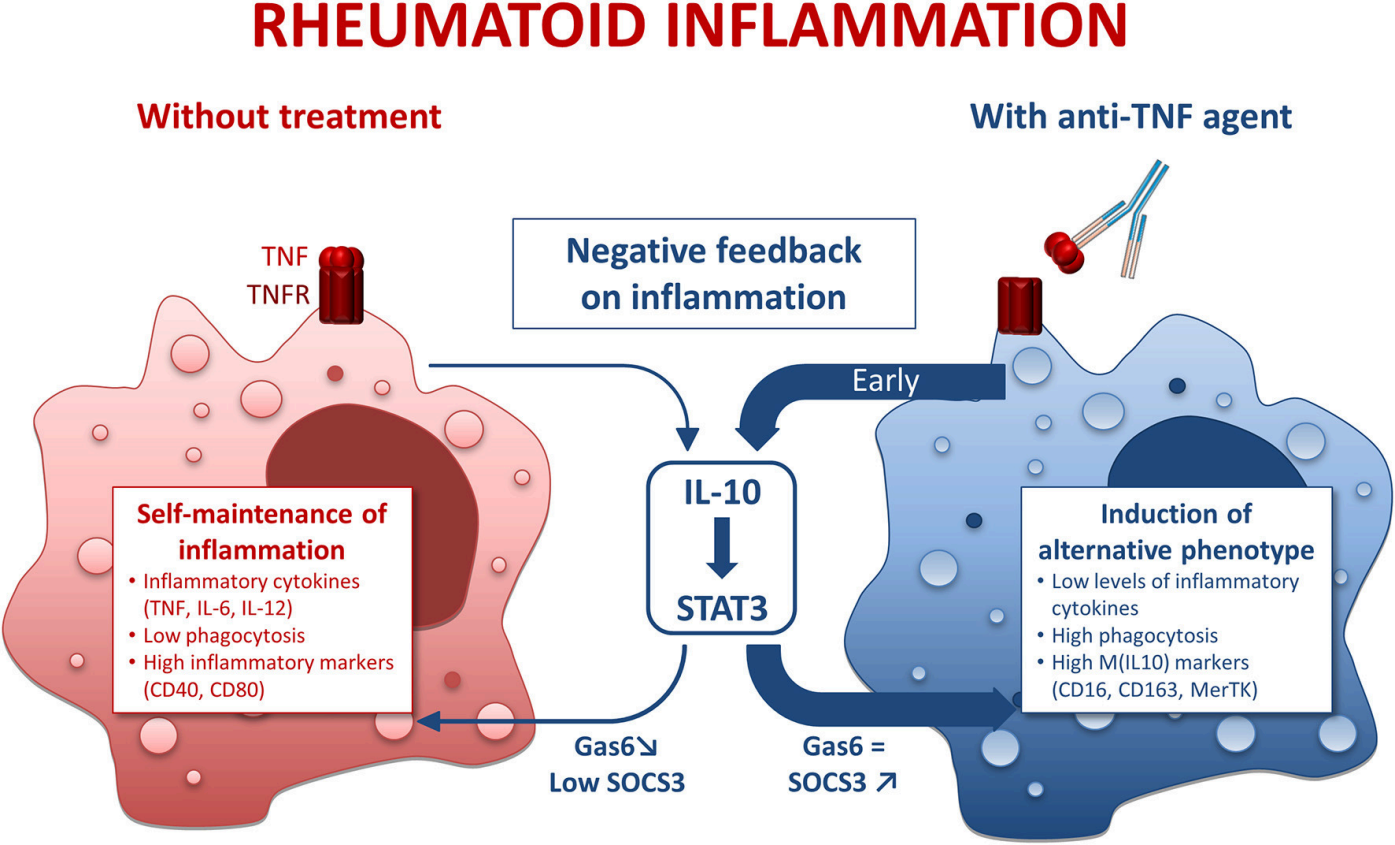
Mode of action of anti-TNF agents in rheumatoid macrophages. IL-10, interleukin 10; STAT3, Signal transducer and activator of transcription 3. Inflammatory conditions such as rheumatoid arthritis (RA) induce macrophages with a pro-inflammatory phenotype. This phenomenon is counterbalanced by a negative feedback involving IL-10. However, in RA the negative feedback on inflammation is overridden, thus leading to a self-maintained inflammation. In the presence of anti-TNF agents, we observed an early and strong negative feedback on inflammation through an IL-10/STAT3 axis resulting in a M(IL10) alternative polarization of macrophages.

Regarding IL-10, we observed a biphasic modulation of IL10 production in the presence of anti-TNF agents: (i) early increase in IL-10, (ii) a decrease at later time-points. Given that IL-10 is a major cytokine of the negative feedback control of inflammation, these findings suggested an implication of IL-10 in the modulation of the M1 MDM phenotype in the presence of anti-TNF agents. This hypothesis was confirmed by the antagonization of anti-TNF agents effects by IL-10 neutralization. We propose that the early increase in IL-10 is a major contributor to the modulation of inflammatory macrophages' polarization by anti-TNF (Figure 6). We observed a decrease in IL-10 at 24 h post-activation. We considered that this decrease was a consequence of the inhibition of the inflammatory milieu provided by the TNF. We have previously shown that CZP, an anti-TNF agent, induce NRF2 activation (21), a marker involved in the anti-inflammatory pathways (45) and IL-10 production (our current study). On the contrary, anti-IL-6R does neither induce NRF2 nor IL-10 production (46–48). Our findings, showing a specific IL-10-dependent modulation of macrophages polarization by anti-TNF agents, thus reflect the differences between bDMARDs on IL-10 regulation. We provide here additional evidence to the concept that a TNF blockade induce a regulatory phenotype in immune cells (49).

Another hallmark of macrophage function is phagocytosis. M1 MDM activated in the presence of anti-TNF agents displayed higher surface expression of phagocytic receptors (CD16, CD163, MerTK) and higher levels of phagocytosis. Clearance of apoptotic bodies, notably involving MerTK, is a critical point for homeostasis and resolution of inflammation. M(IL10) polarization potentiates clearance of apoptotic bodies through MerTK (26). Our data emphasize that anti-TNF agents orientate inflammatory macrophages toward a pro-resolving phenotype.

In addition to the increase in phagocytosis by anti-TNF agents, we observed an increase in MerTK surface expression, and in *gas6* mRNA. MerTK is, like other TAM receptors, involved in negative regulation of TLR-mediated inflammation by antigen presenting (28). MerTK expression is linked to M(IL-10) alternative polarization. GAS6, a TAM receptor ligand, has been shown to inhibit TNF, IL-6, and IL-1 secretion in human monocytes (50). Overexpression of TAM ligands is known to increase SOCS3 levels and contributes to dampen murine arthritis (29). Moreover, IL-10 favors efferocytosis *via* MerTK/Gas6, and Gas6 is known to be able to drive anti-inflammatory effects through IL-10 induction (26). Our results emphasize an upregulation of MerTK, *gas6*, and phagocytosis in the presence of anti-TNF agents, consistent with an upregulation of the negative feedback control of inflammation by TNF blockade involving MerTK and its counterparts.

IL-10 is known to signal through Jak1/STAT3 pathway. We have shown that modulation of macrophages' polarization by anti-TNF agents was IL-10/STAT3 dependent. The implication of the IL-10/STAT3 axis may be related to the increase in IL-10 (mRNA and protein) at early time-points.

In conclusion, we showed that anti-TNF agents not only inhibit *in vitro* inflammatory functions of macrophages, but also favor pro-resolving features of macrophages specifically involving the IL-10/STAT3 axis. Given that patients of our study have been treated with different DMARDs or MTX schedules, the conclusions may not apply to all patients. Among those concerned, such a shift in macrophage polarization in inflammatory context is expected to contribute to the clinical benefit of anti-TNF agents in RA.

## Ethics Statement

This study was carried out in accordance with the recommendations of the Declaration of Helsinki. All subjects gave written informed consent in accordance with the Declaration of Helsinki. The protocol was approved by the local ethics committee: CHU Toulouse—BioTOUL DC 2016-2804.

## Author Contributions

YD, AC, and J-LD study conception and design. YD, BR, MB, AR-W, and AC acquisition of data. YD, BR, MB, J-FB, AR-W, AC, and J-LD analysis and interpretation of data. All authors were involved in drafting the article or revising it critically for important intellectual content, and all authors approved the final version to be published.

### Conflict of Interest Statement

YD received a fellowship from la Société Française de Rhumatologie. AC and J-LD received a Passerelle Grant from Pfizer France. The remaining authors declare that the research was conducted in the absence of any commercial or financial relationships that could be construed as a potential conflict of interest.

## References

[1] Gordon S Martinez FO. Alternative activation of macrophages: mechanism and functions. Immunity (2010) 32:593–604. 10.1016/j.immuni.2010.05.007 20510870

[2] Martinez FO Gordon S. The M1 and M2 paradigm of macrophage activation: time for reassessment. F1000Prime Rep. (2014) 6:13. 10.12703/P6-13 24669294 PMC3944738

[3] Xue J Schmidt SV Sander J Draffehn A Krebs W Quester I. Transcriptome-based network analysis reveals a spectrum model of human macrophage activation. Immunity (2014) 40:274–88. 10.1016/j.immuni.2014.01.006 24530056 PMC3991396

[4] Ambarus CA Noordenbos T de Hair MJ Tak PP Baeten DL. Intimal lining layer macrophages but not synovial sublining macrophages display an IL-10 polarized-like phenotype in chronic synovitis. Arthritis Res Ther. (2012) 14:R74. 10.1186/ar3796 22494514 PMC3446447

[5] Balsa A Dixey J Sansom DM Maddison PJ Hall ND. Differential expression of the costimulatory molecules B7.1 (CD80) and B7.2 (CD86) in rheumatoid synovial tissue. Br J Rheumatol. (1996) 35:33–7. 10.1093/rheumatology/35.1.33 8624620

[6] Soler Palacios B Estrada-Capetillo L Izquierdo E Criado G Nieto C Municio C Macrophages from the synovium of active rheumatoid arthritis exhibit an activin A-dependent pro-inflammatory profile. J Pathol. (2015) 235:515–26. 10.1002/path.4466 25319955

[7] Chu CQ Field M Feldmann M Maini RN. Localization of tumor necrosis factor alpha in synovial tissues and at the cartilage-pannus junction in patients with rheumatoid arthritis. Arthritis Rheum. (1991) 34:1125–32. 10.1002/art.1780340908 1930331

[8] Szekanecz Z Koch AE. Successes and failures of chemokine-pathway targeting in rheumatoid arthritis. Nat Rev Rheumatol. (2016) 12:5–13. 10.1038/nrrheum.2015.157 26607389

[9] Elshabrawy HA Chen Z Volin MV Ravella S Virupannavar S Shahrara S. The pathogenic role of angiogenesis in rheumatoid arthritis. Angiogenesis (2015) 18:433–48. 10.1007/s10456-015-9477-2 26198292 PMC4879881

[10] Kayakabe K Kuroiwa T Sakurai N Ikeuchi H Kadiombo AT Sakairi T. Interleukin-6 promotes destabilized angiogenesis by modulating angiopoietin expression in rheumatoid arthritis. Rheumatology (Oxford) (2012) 51:1571–9. 10.1093/rheumatology/kes093 22596210

[11] Leibovich SJ Polverini PJ Shepard HM Wiseman DM Shively V Nuseir N. Macrophage-induced angiogenesis is mediated by tumour necrosis factor-alpha. Nature (1987) 329:630–2. 10.1038/329630a0 2443857

[12] Maruotti N Annese T Cantatore FP Ribatti D. Macrophages and angiogenesis in rheumatic diseases. Vasc Cell (2013) 5:11. 10.1186/2045-824X-5-11 23725043 PMC3680215

[13] Miesel R Murphy MP Kroger H. Enhanced mitochondrial radical production in patients which rheumatoid arthritis correlates with elevated levels of tumor necrosis factor alpha in plasma. Free Radic Res. (1996) 25:161–9. 10.3109/10715769609149921 8885334

[14] Lundy SK Sarkar S Tesmer LA Fox DA. Cells of the synovium in rheumatoid arthritis. T lymphocytes. Arthritis Res Ther. (2007) 9:202. 10.1186/ar2107 17306038 PMC1860060

[15] Roberts CA Dickinson AK Taams LS. The interplay between monocytes/macrophages and CD4(+) T cell subsets in rheumatoid arthritis. Front Immunol. (2015) 6:571. 10.3389/fimmu.2015.00571 26635790 PMC4652039

[16] Bartok B Firestein GS. Fibroblast-like synoviocytes: key effector cells in rheumatoid arthritis. Immunol Rev. (2010) 233:233–55. 10.1111/j.0105-2896.2009.00859.x 20193003 PMC2913689

[17] Takayanagi H. Osteoimmunology: shared mechanisms and crosstalk between the immune and bone systems. Nat Rev Immunol. (2007) 7:292–304. 10.1038/nri2062 17380158

[18] Arnoux F Mariot C Peen E Lambert NC Balandraud N Roudier J. Peptidyl arginine deiminase immunization induces anticitrullinated protein antibodies in mice with particular MHC types. Proc Natl Acad Sci USA. (2017) 114:E10169–77. 10.1073/pnas.1713112114 29109281 PMC5703315

[19] Vossenaar ER. Expression and activity of citrullinating peptidylarginine deiminase enzymes in monocytes and macrophages. Ann Rheum Dis. (2004) 63:373–81. 10.1136/ard.2003.012211 15020330 PMC1754951

[20] Haringman JJ Gerlag DM Zwinderman AH Smeets TJ Kraan MC Baeten D. Synovial tissue macrophages: a sensitive biomarker for response to treatment in patients with rheumatoid arthritis. Ann Rheum Dis. (2005) 64:834–8. 10.1136/ard.2004.029751 15576415 PMC1755544

[21] Boyer JF Baron M Constantin A Degboe Y Cantagrel A Davignon JL. Anti-TNF certolizumab pegol induces antioxidant response in human monocytes via reverse signaling. Arthritis Res Ther. (2016) 18:56. 10.1186/s13075-016-0955-8 26932562 PMC4774095

[22] Catrina AI Trollmo C af Klint E Engstrom M Lampa J Hermansson Y Evidence that anti-tumor necrosis factor therapy with both etanercept and infliximab induces apoptosis in macrophages, but not lymphocytes, in rheumatoid arthritis joints: extended report. Arthritis Rheum. (2005) 52:61–72. 10.1002/art.20764 15641091

[23] Smeets TJ Kraan MC van Loon ME Tak PP Tumor necrosis factor alpha blockade reduces the synovial cell infiltrate early after initiation of treatment, but apparently not by induction of apoptosis in synovial tissue. Arthritis Rheum. (2003) 48:2155–62. 10.1002/art.11098 12905468

[24] Murray PJ Allen JE Biswas SK Fisher EA Gilroy DW Goerdt S. Macrophage activation and polarization: nomenclature and experimental guidelines. Immunity (2014) 41:14–20. 10.1016/j.immuni.2014.06.008 25035950 PMC4123412

[25] Niemand C Nimmesgern A Haan S Fischer P Schaper F Rossaint R. Activation of STAT3 by IL-6 and IL-10 in primary human macrophages is differentially modulated by suppressor of cytokine signaling 3. J Immunol. (2003) 170:3263–72. 10.4049/jimmunol.170.6.3263 12626585

[26] Zizzo G Hilliard BA Monestier M Cohen PL. Efficient clearance of early apoptotic cells by human macrophages requires M2c polarization and MerTK induction. J Immunol. (2012) 189:3508–20. 10.4049/jimmunol.1200662 22942426 PMC3465703

[27] McCormick SM Heller NM. Regulation of macrophage, dendritic cell, and microglial phenotype and function by the SOCS proteins. Front Immunol. (2015) 6:549. 10.3389/fimmu.2015.00549 26579124 PMC4621458

[28] Lu Q Lemke G. Homeostatic regulation of the immune system by receptor tyrosine kinases of the Tyro 3 family. Science (2001) 293:306–11. 10.1126/science.1061663 11452127

[29] van den Brand BT Abdollahi-Roodsaz S Vermeij EA Bennink MB Arntz OJ Rothlin CV Therapeutic efficacy of Tyro3, Axl, and Mer tyrosine kinase agonists in collagen-induced arthritis. Arthritis Rheum. (2013) 65:671–80. 10.1002/art.37786 23203851 PMC3582862

[30] Yasukawa H Ohishi M Mori H Murakami M Chinen T Aki D. IL-6 induces an anti-inflammatory response in the absence of SOCS3 in macrophages. Nat Immunol. (2003) 4:551–6. 10.1038/ni938 12754507

[31] Aeberli D Kamgang R Balani D Hofstetter W Villiger PM Seitz M. Regulation of peripheral classical and non-classical monocytes on infliximab treatment in patients with rheumatoid arthritis and ankylosing spondylitis. RMD Open (2016) 2:e000079. 10.1136/rmdopen-2015-000079 26819749 PMC4716562

[32] Huang QQ Birkett R Doyle R Shi B Roberts EL Mao Q. The role of macrophages in the response to TNF inhibition in experimental arthritis. J Immunol. (2018) 200:130–8. 10.4049/jimmunol.1700229 29150565 PMC5736443

[33] Vos AC Wildenberg ME Arijs I Duijvestein M Verhaar AP de Hertogh G. Regulatory macrophages induced by infliximab are involved in healing *in vivo* and *in vitro*. Inflamm Bowel Dis. (2012) 18:401–8. 10.1002/ibd.21818 21936028

[34] Munn DH Beall AC Song D Wrenn RW Throckmorton DC. Activation-induced apoptosis in human macrophages: developmental regulation of a novel cell death pathway by macrophage colony-stimulating factor and interferon gamma. J Exp Med. (1995) 181:127–36. 10.1084/jem.181.1.127 7806999 PMC2191830

[35] Xaus J Comalada M Valledor AF Lloberas J Lopez-Soriano F Argiles JM. LPS induces apoptosis in macrophages mostly through the autocrine production of TNF-alpha. Blood (2000) 95:3823–31. 10845916

[36] Nesbitt A Fossati G Bergin M Stephens P Stephens S Foulkes R. Mechanism of action of certolizumab pegol (CDP870): *in vitro* comparison with other anti-tumor necrosis factor alpha agents. Inflamm Bowel Dis. (2007) 13:1323–32. 10.1002/ibd.20225 17636564

[37] Herenius MM Thurlings RM Wijbrandts CA Bennink RJ Dohmen SE Voermans C. Monocyte migration to the synovium in rheumatoid arthritis patients treated with adalimumab. Ann Rheum Dis. (2011) 70:1160–2. 10.1136/ard.2010.141549 21345816 PMC3086080

[38] Hashimoto S Suzuki T Dong HY Yamazaki N Matsushima K. Serial analysis of gene expression in human monocytes and macrophages. Blood (1999) 94:837–44. 10419873

[39] Lacey DC Achuthan A Fleetwood AJ Dinh H Roiniotis J Scholz GM. Defining GM-CSF- and macrophage-CSF-dependent macrophage responses by *in vitro* models. J Immunol. (2012) 188:5752–65. 10.4049/jimmunol.1103426 22547697

[40] Campbell IK Rich MJ Bischof RJ Hamilton JA. The colony-stimulating factors and collagen-induced arthritis: exacerbation of disease by M-CSF and G-CSF and requirement for endogenous M-CSF. J Leukoc Biol. (2000) 68:144–50. 10914502

[41] Clavel C Ceccato L Anquetil F Serre G Sebbag M. Among human macrophages polarised to different phenotypes, the M-CSF-oriented cells present the highest pro-inflammatory response to the rheumatoid arthritis-specific immune complexes containing ACPA. Ann Rheum Dis. (2016) 75:2184–91. 10.1136/annrheumdis-2015-208887 27009917

[42] Garcia S Hartkamp LM Malvar-Fernandez B van Es IE Lin H Wong J. Colony-stimulating factor (CSF) 1 receptor blockade reduces inflammation in human and murine models of rheumatoid arthritis. Arthritis Res Ther. (2016) 18:75. 10.1186/s13075-016-0973-6 27036883 PMC4818474

[43] Hayder M Poupot M Baron M Nigon D Turrin CO Caminade AM. A phosphorus-based dendrimer targets inflammation and osteoclastogenesis in experimental arthritis. Sci Transl Med. (2011) 3:81ra35. 10.1126/scitranslmed.3002212 21543721

[44] Ambarus CA Krausz S van Eijk M Hamann J Radstake TR Reedquist KA. Systematic validation of specific phenotypic markers for *in vitro* polarized human macrophages. J Immunol Methods (2012) 375:196–206. 10.1016/j.jim.2011.10.013 22075274

[45] Mills EL Ryan DG Prag HA Dikovskaya D Menon D Zaslona Z. Itaconate is an anti-inflammatory metabolite that activates Nrf2 via alkylation of KEAP1. Nature (2018) 556:113–7. 10.1038/nature25986 29590092 PMC6047741

[46] Dijkgraaf EM Heusinkveld M Tummers B Vogelpoel LT Goedemans R Jha V. Chemotherapy alters monocyte differentiation to favor generation of cancer-supporting M2 macrophages in the tumor microenvironment. Cancer Res. (2013) 73:2480–92. 10.1158/0008-5472.CAN-12-3542 23436796

[47] Matsuoka Y Nakayama H Yoshida R Hirosue A Nagata M Tanaka T. IL-6 controls resistance to radiation by suppressing oxidative stress via the Nrf2-antioxidant pathway in oral squamous cell carcinoma. Br J Cancer (2016) 115:1234–44. 10.1038/bjc.2016.327 27736845 PMC5104896

[48] Noack M Ndongo-Thiam N Miossec P. Evaluation of anti-inflammatory effects of steroids and arthritis-related biotherapies in an *in vitro* coculture model with immune cells and synoviocytes. Front Immunol. (2016) 7:509. 10.3389/fimmu.2016.00509 27909436 PMC5112278

[49] Roberts CA Durham LE Fleskens V Evans HG Taams LS. TNF blockade maintains an IL-10(+) phenotype in human effector CD4(+) and CD8(+) T cells. Front Immunol. (2017) 8:157. 10.3389/fimmu.2017.00157 28261215 PMC5309392

[50] Alciato F Sainaghi PP Sola D Castello L Avanzi GC. TNF-alpha, IL-6, and IL-1 expression is inhibited by GAS6 in monocytes/macrophages. J Leukoc Biol. (2010) 87:869–75. 10.1189/jlb.0909610 20103767

