# Supplementary material for: Polarization of Rheumatoid Macrophages by TNF Targeting Through an IL-10/STAT3 Mechanism
Source: Front Immunol. 2019 Jan 18;10:3. doi: 10.3389/fimmu.2019.00003 (PMC6345709; doi:10.3389/fimmu.2019.00003)
Supplement: Supplementary file 1 [file Data_Sheet_1.PDF]

ONLINE SUPPLEMENTAL FILE

Polarization of Rheumatoid Macrophages by TNF Targeting through an IL-10/STAT3 Mechanism.

Yannick Degboé, Benjamin Rauwel, Michel Baron, Jean-Frédéric Boyer, Adeline Ruyssen-Witrand, Arnaud Constantin, Jean-Luc Davignon.

Page 2. Supplemental Figure S1. Anti-TNF agents favor alternative polarization of macrophages

Page 4. Supplemental Figure S2. Effect of bDMARDs on surface markers on inflammatory macrophages.

Page 5. Supplemental Figure S3. Impact of treatment regimen at inclusion, on the *in vitro* modulation of polarization markers by bDMARDs.

Page 7. Supplemental Figure S4. The modulation of macrophage polarization markers by anti-TNF agents is Fc-independent.

Page 8. Supplemental Table S1. Anti-TNF agents decrease macrophage inflammatory cytokines associated with inflammatory activation.

Page 9. Supplemental Figure S5. Effect of anti-TNF agents on TNF detection and secretion.

Page 10. Supplemental Figure S6. Etanercept modulates macrophage function by inhibiting TNF production.

Page 11. Supplemental Figure S7. Assessment of phagocytosis

Page 12. Supplemental Figure S8. The impact of anti-TNF agents on macrophage polarization involves IL-10.

Page 13. Supplemental Figure 9. Comparison of the effects of STAT3 inhibitors and anti-IL-10 neutralizing monoclonal antibody

RESULTS

Supplemental Figure S1. Anti-TNF agents favor alternative polarization of macrophages

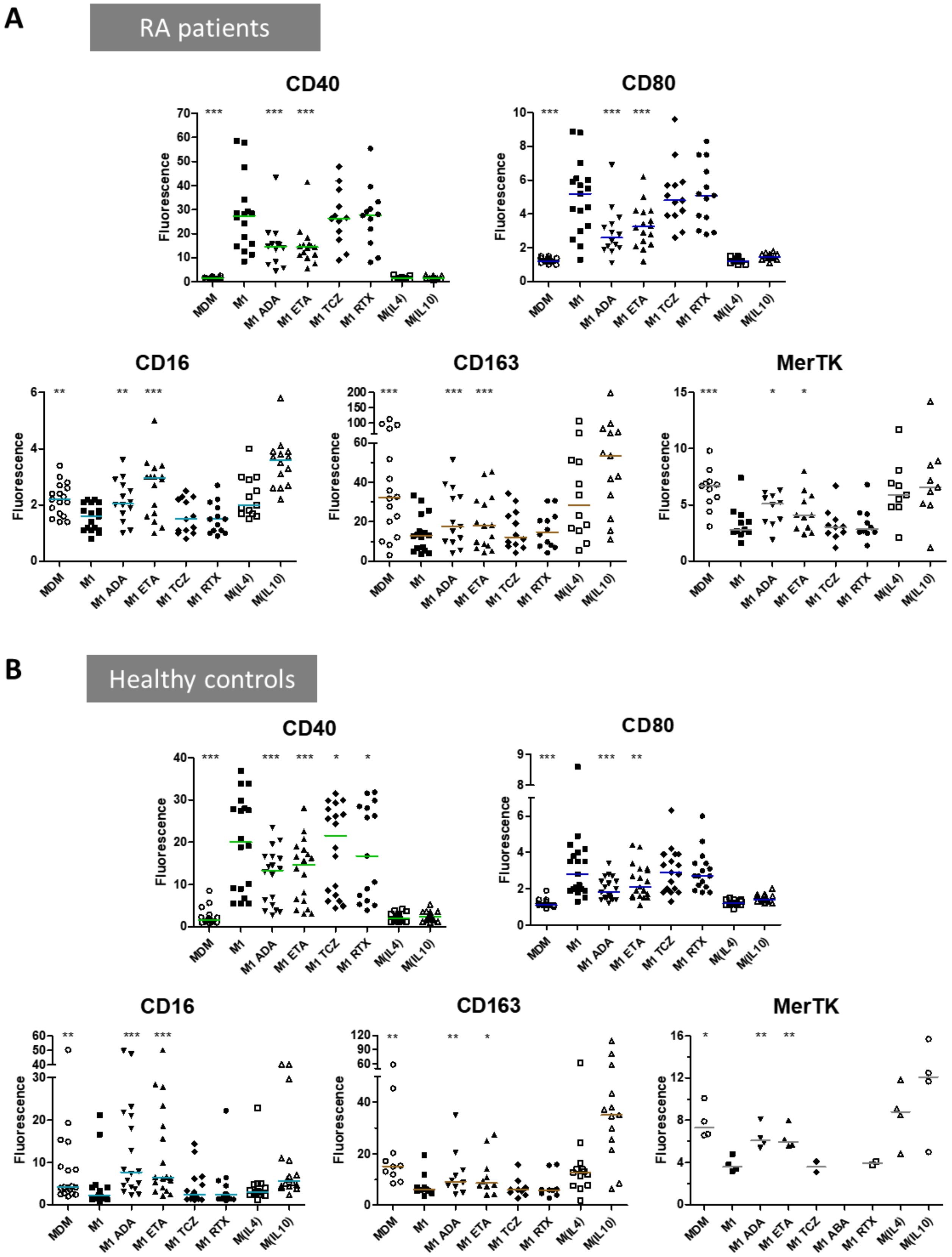

MDM: monocyte-derived macrophages. M1: pro-inflammatory macrophages activated by LPS + IFNγ. M2(IL4): alternative macrophages activated by IL-4. M2(IL10): alternative macrophages activated by IL-10. ADA: adalimumab. ETA: etanercept TCZ: tocilizumab. RTX: rituximab. RA: rheumatoid arthritis.
(A, B) *Monocyte-derived macrophages were activated for 24h (as M1, M2(IL4) and M2(IL10) with or without the indicated bDMARD. Surface polarization markers on macrophages from up to 16 RA patients* ***(A)*** *and 20 healthy controls* ***(B)*** *were analyzed by flow cytometry. Non-activated MDM and M1 MDM activated in the presence of bDMARDs were compared to M1 MDM. ***: p value <0.001, **: 0.001≤p< 0.01, *: 0.01≤p< 0.05 (Student paired t-test or Wilcoxon matched pairs test).*

Supplemental Figure S2. Effect of bDMARDs on surface markers on inflammatory macrophages.

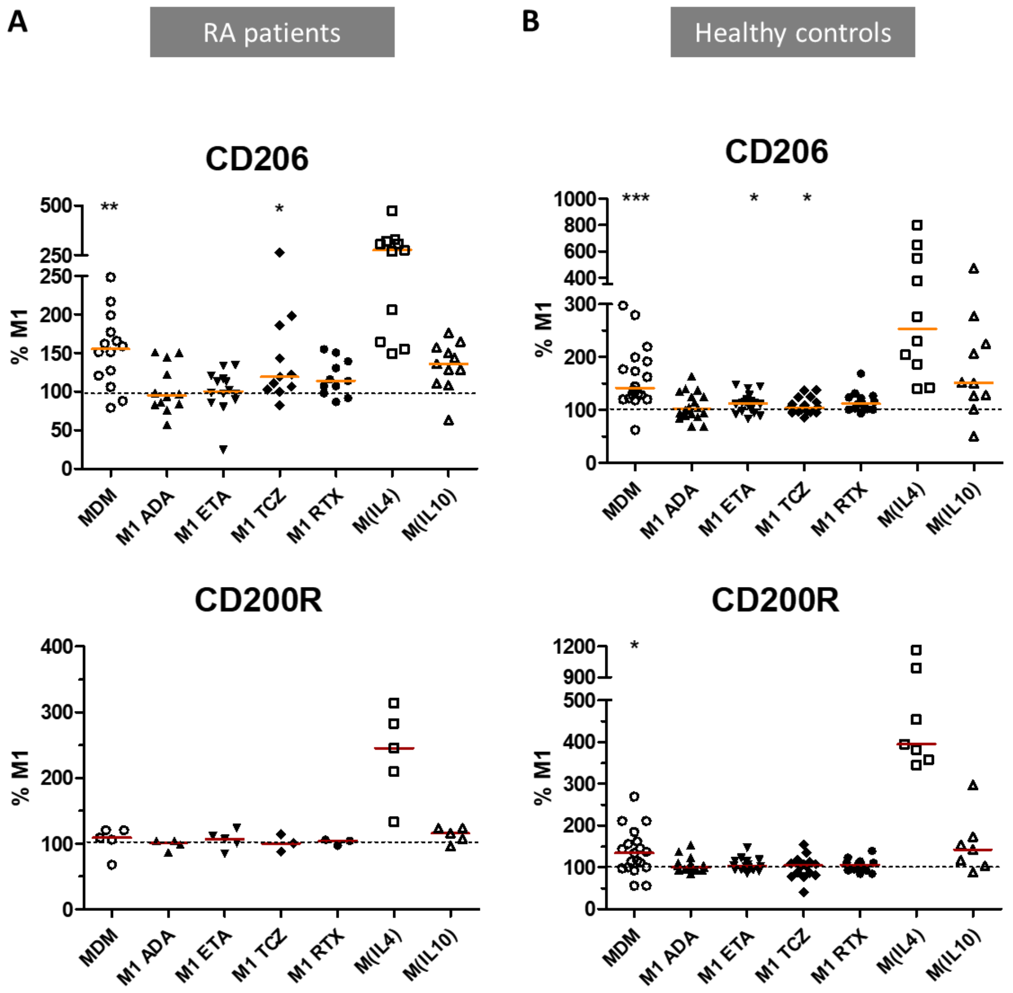

MDM: monocyte-derived macrophages. M1: pro-inflammatory macrophages activated by LPS + IFNγ. M2(IL4): alternative macrophages activated by IL-4. M2(IL10): alternative macrophages activated by IL-10. ADA: adalimumab. ETA: etanercept. TCZ: tocilizumab. RTX: rituximab. RA: rheumatoid arthritis.

(A, B) *Monocyte-derived macrophages were activated for 24h (as M1, M2(IL4) and M2(IL10) with or without the indicated bDMARD. Surface polarization markers on macrophages from up to 16 RA patients* ***(A)*** *and 20 healthy controls* ***(B)*** *were analyzed by flow cytometry. Non-activated MDM and M1 MDM activated in the presence of bDMARDs were compared to M1 MDM. ***: p value <0.001, **: 0.001≤p< 0.01, *: 0.01≤p< 0.05 (Student paired t-test or Wilcoxon matched pairs test).*

Supplemental Figure S3. Impact of treatment regimen at inclusion on the *in vitro* modulation of polarization markers by bDMARDs.

A

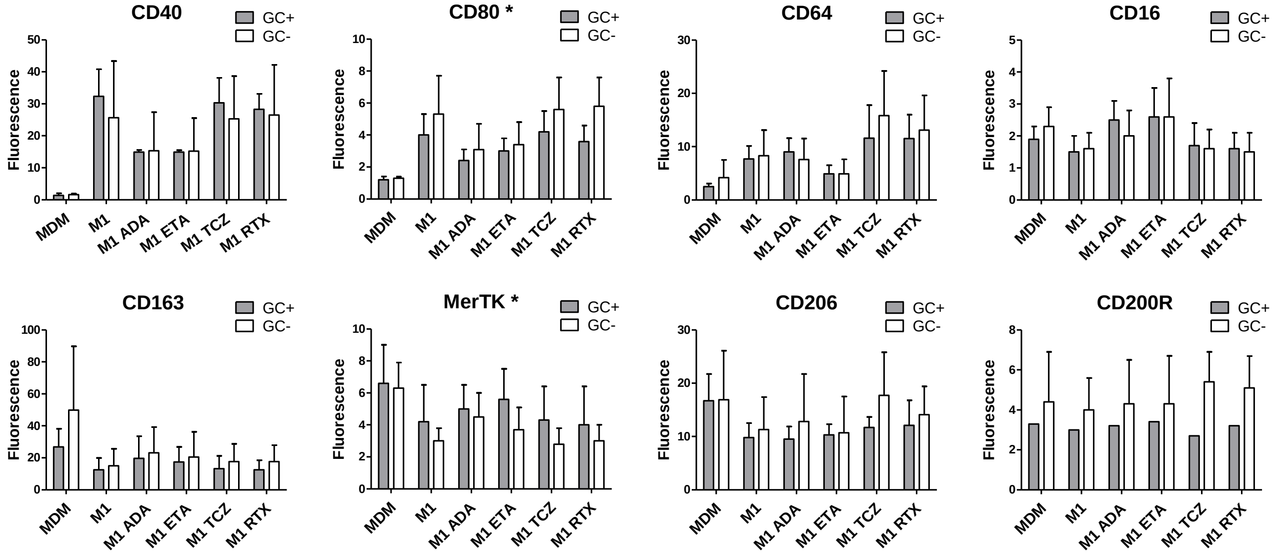

*: GC use accounts for: (i) 5.7% of the total variance of CD80 expression ($p=0.0053$), (ii) 7.6% of the total variance of MerTK expression ($p=0.0146$)

B

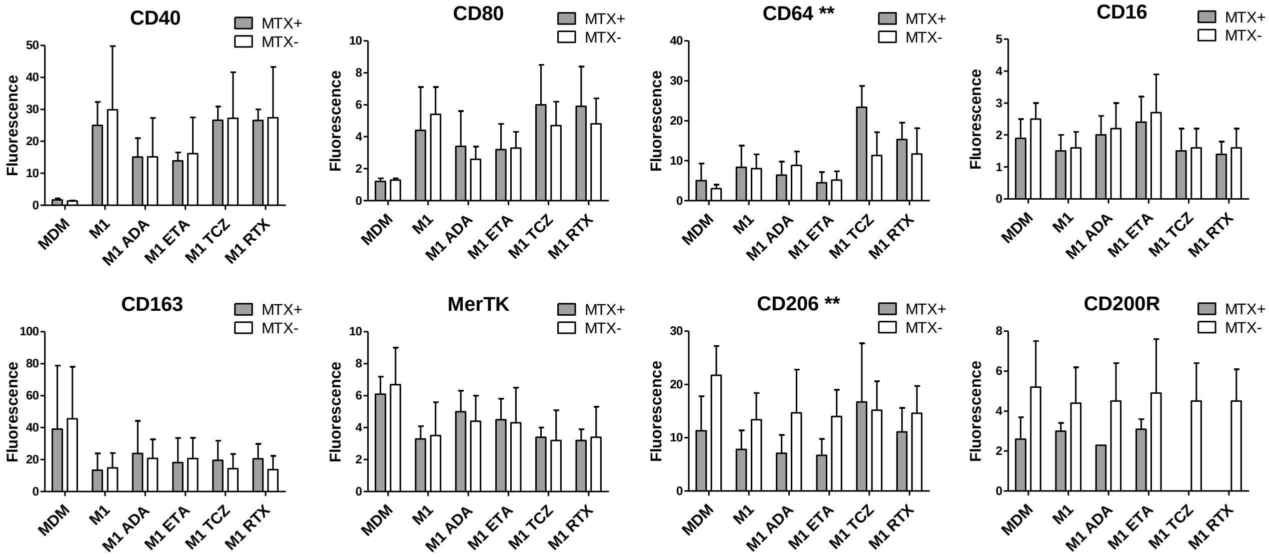

**: MTX use accounts for: (i) 16.0% of the total variance of CD206 expression ($p<0.0001$), (ii) 3.4% of the total variance of CD64 expression ($p=0.0168$)

C

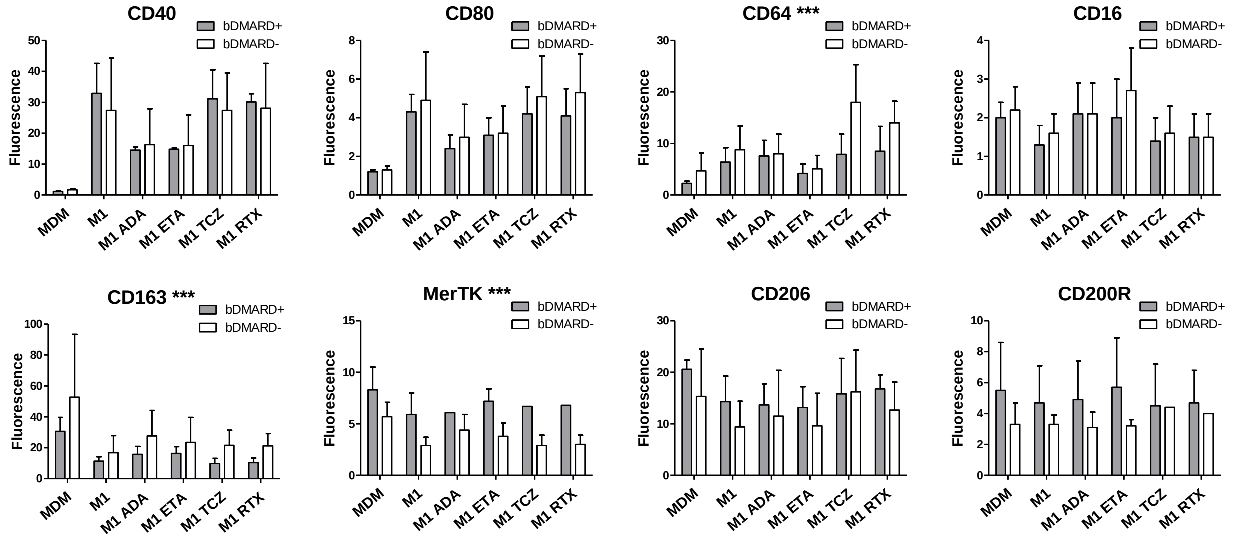

***: bDMARD use accounts for: (i) 6.7% of the total variance of CD163 expression (p=0.0225), (ii) 41.0% of the total variance of MerTK expression (p<0.0001), (iii) 9.9% of the total variance of CD64 (p=0.0015)

MDM: monocyte-derived macrophages. M1: pro-inflammatory macrophages activated by LPS + IFNγ. GC: steroids use. MTX: methotrexate use. bDMARD: biologics use. ADA: adalimumab. ETA: etanercept. TCZ: tocilizumab. RTX: rituximab. RA: rheumatoid arthritis. Representation of membrane expression of polarization markers according background treatment regimen i.e. GC ***(A)****, MTX* ***(B)****, and bDMARD* ***(C)****. The influence was assessed by a two-way ANOVA test.*

Supplemental Figure S4. The modulation of macrophage polarization markers by anti-TNF agents is Fc-independent.

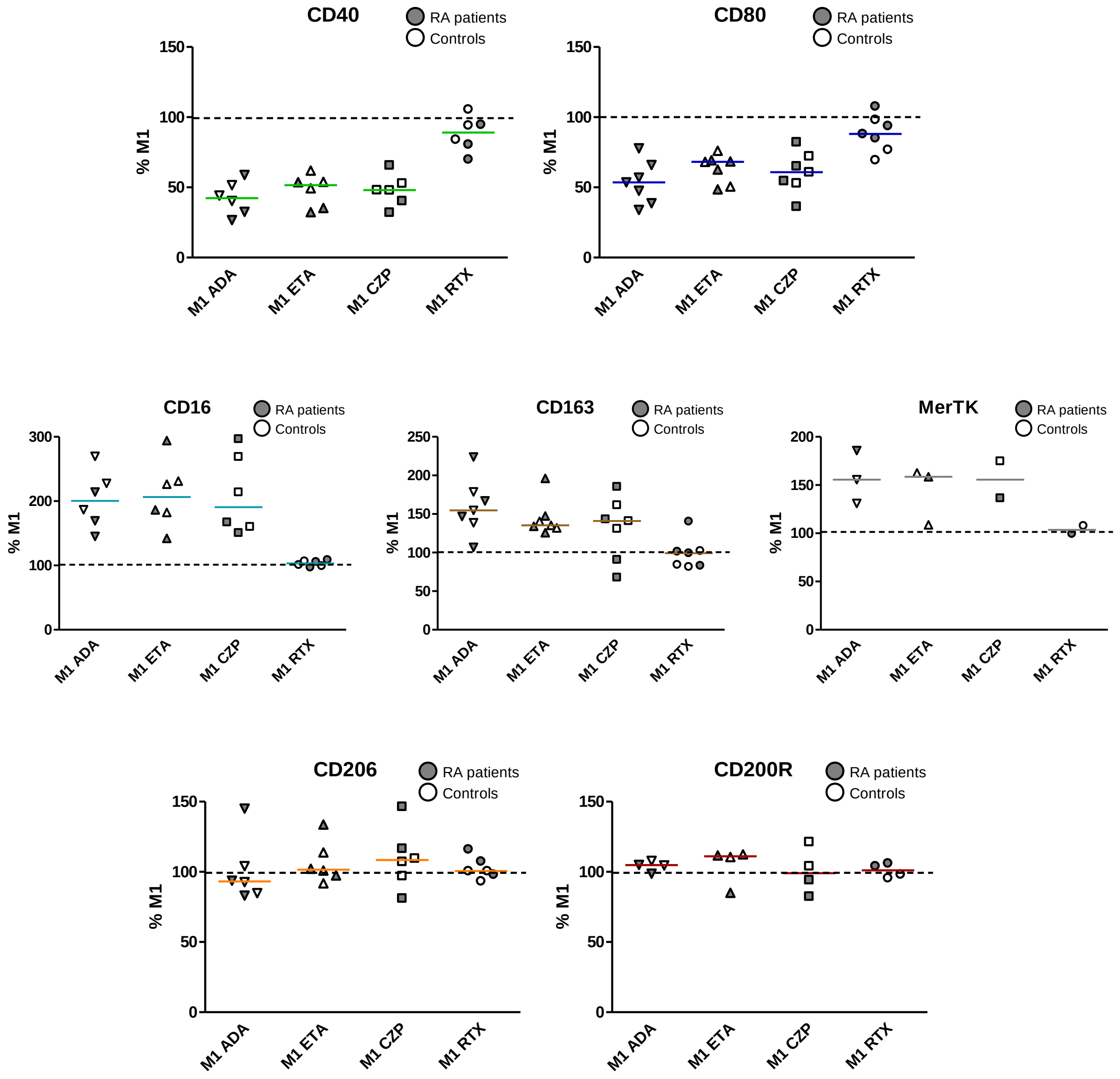

ADA: adalimumab. ETA: etanercept. CZP: certolizumab. RTX: rituximab. M1: pro-inflammatory macrophages activated by LPS + IFNγ. Macrophages from RA patients (grey symbols) and healthy controls (open symbols) were obtained after 5 days of differentiation with M-CSF and then activated for 24h as M1 (LPS + IFNγ) with or without the indicated bDMARD. Surface markers were assessed by flow cytometry.

Supplemental Table S1. Anti-TNF agents decrease macrophage inflammatory cytokines associated with inflammatory activation.

A RA patients

Median (IQR) pg/mL	IL6	IL12	TNF
**Non activated MDM**	17.4 (8.9 – 86.4)	0.0 (0.0 – 1.6)	3.4 (0.0 – 16.1)
**M1**	**6904.0 (2955.0 – 11337.0)**	**26.3 (9.6 – 138.9)**	**3936.0 (1715.0 – 8700.0)**
**M1 ADA**	3702.0 (2122.0 – 4334.0)	17.0 (6.1 – 30.8)	19.9 (13.8 – 33.7)
**M1 ETA**	2238.0 (1354.0 – 6168.0)	9.9 (6.2 – 43.5)	4668.0 (3219.0 – 8406.0)
**M1 TCZ**	6309.0 (3620.0 – 8265.0)	34.6 (15.6 – 94.6)	4402.0 (2756.0 – 6023.0)
**M1 RTX**	3592.0 (2515.0 – 7315.0)	41.2 (14.3 – 91.9)	4840.0 (2199.0 – 6818.0)

B Healthy controls

Median (IQR) pg/mL	IL6	IL12	TNF
**Non activated MDM**	5.1 (1.9 – 22.2)	0.0 (0.0 – 0.7)	0.0 (0.0 – 9.1)
**M1**	**2214.0 (946.4 – 3265.0)**	**14.1 (9.1 – 28.8)**	**1318.0 (898.7 – 4684.0)**
**M1 ADA**	1317.0 (574.1 – 2140.0)	3.4 (1.0 – 4.5)	5.6 (1.1 – 12.8)
**M1 ETA**	833.8 (542.2 – 2607.0)	4.7 (2.4 – 6.4)	1421.0 (516.9 – 2419.0)
**M1 TCZ**	713.1 (620.9 – 2244.0)	12.6 (8.7 – 19.4)	976.7 (656.1 – 2396.0)
**M1 RTX**	1048.0 (716.2 – 2416.0)	12.2 (6.2 – 21.6)	1099.0 (1019.0 – 3519.0)

*MDM: monocyte-derived macrophages. M1: pro-inflammatory macrophages activated by LPS + IFNγ. M2(IL4): alternative macrophages activated by IL-4. M2(IL10): alternative macrophages activated by IL-10. RA: rheumatoid arthritis. ADA: adalimumab. ETA: etanercept. TCZ: tocilizumab. RTX: rituximab. RA: rheumatoid arthritis. Cytokine secretion was measured by cytometric bead array in cell culture supernatants, after 24h of activation of macrophages from RA patients **(A)** and healthy controls **(B)**.*

Supplemental Figure S5. Effect of anti-TNF agents on TNF detection and secretion.

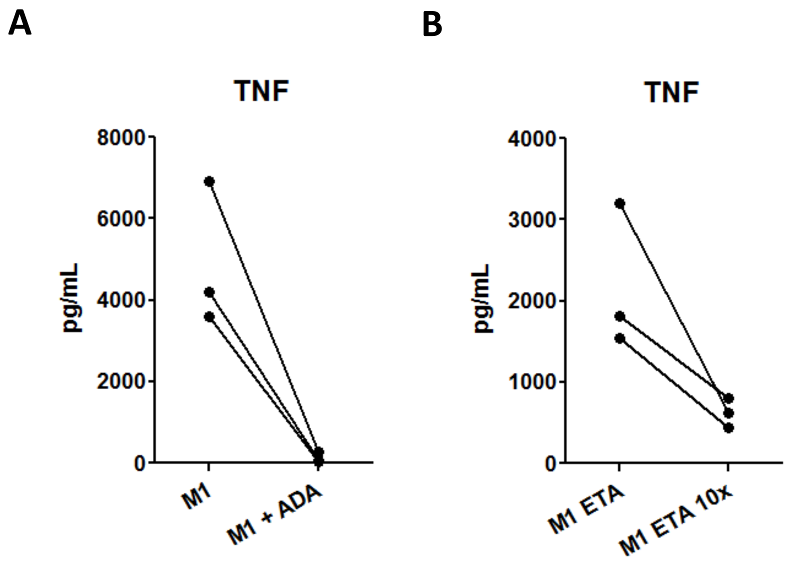

(A) *TNF detection by cytometric bead array in M1 macrophages supernatant was competed by ADA 100 µg/mL (3 healthy controls).* ***(B)*** *Influence of ETA on TNF secretion by inflammatory macrophages was assessed at 10 µg/mL and at a higher dose of ETA (100 µg/mL; 3 healthy controls).*

Supplemental Figure S6. Etanercept modulates macrophage function by inhibiting TNF production.

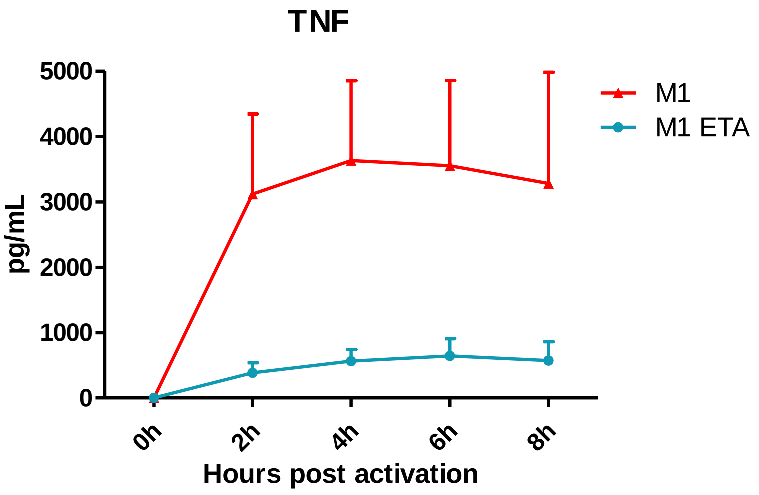

Early TNF secretion was measured in cell culture supernatants of treated (etanercept) and untreated M1 macrophages by cytometric bead array (3 healthy controls).

Supplemental Figure S7. Assessment of Phagocytosis

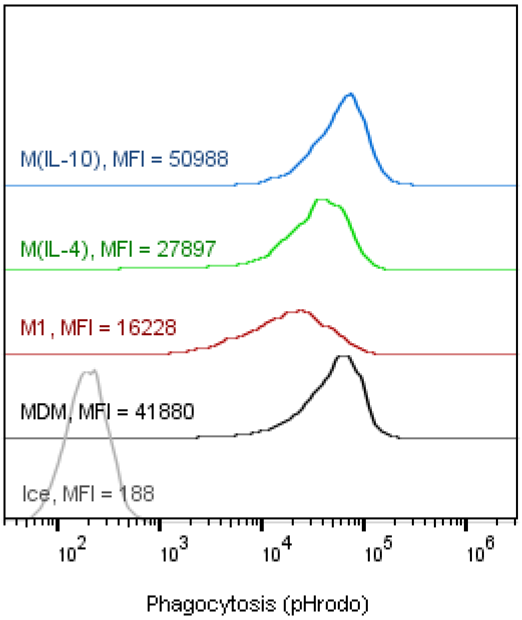

Phagocytosis of green-labelled E.coli particles was assessed by flow cytometry. Representative data from one experiment.

Supplemental Figure S8. The impact of anti-TNF agents on macrophage polarization involves IL-10.

A

IL-10 Median (IQR) pg/mL	Non activated MDM	M1	M1 ADA	M1 ETA	M1 TCZ	M1 RTX
**RA patients**	12,1<br>(9.8 – 22.4)	**2741.0**<br>**(1322.0 –4381.0)**	719.3<br>(528.6 – 805.3)	505.7<br>(354.2 – 633.7)	1873.0<br>(1111.0– 2541.0)	4307.0<br>(1726.0– 4868.0)
**Healthy controls**	6.6<br>(5.7 – 10.6)	**986.7**<br>**(471.8 – 1195.0)**	205.4<br>(141.3 – 242.1)	190.7<br>(154.9 – 261.6)	746.0<br>(673.0 – 1033.0)	563.1<br>(348.0 – 850.1)

B

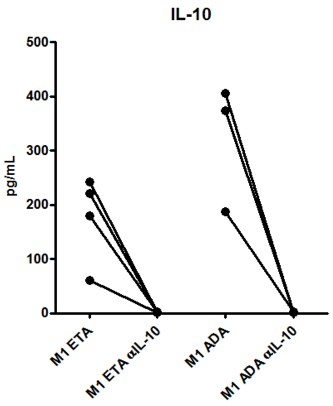

C

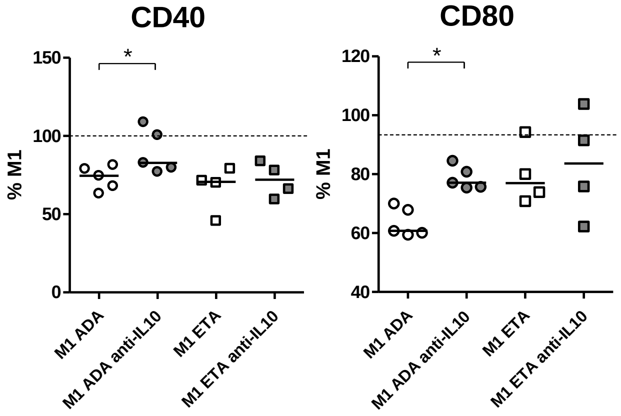

MDM: monocyte-derived macrophages. M1: pro-inflammatory macrophages activated by LPS + IFNγ. ETA: etanercept. ADA: adalimumab. TCZ: tocilizumab. RTX: rituximab. ***(A)*** *IL-10 secretion was measured in cell culture supernatants, after 1 day of M1 activation in the presence of the indicated bDMARD, using cytometric bead array (10 RA patients and 11 healthy donors).* ***(B)*** *Quantification of IL-10 secretion after IL-10 neutralization (4 healthy donors).* ***(C)*** *Surface marker expression assessed by flow cytometry, in M1 MDM, treated or not with the indicated anti-TNF, in the presence or not of IL-10 neutralizing antibody. Results are standardized to M1 MDM treated by anti-TNF agent (RA patients and healthy donors). Non-activated MDM and M1 MDM cultivated in the presence of the indicated bDMARDs were compared to M1 MDM *: 0.01≤p< 0.05 (Student paired t-test or Wilcoxon matched pairs test).*

Supplemental Figure 9. Comparison of the effects of STAT3 inhibitors and anti-IL-10 neutralizing monoclonal antibody

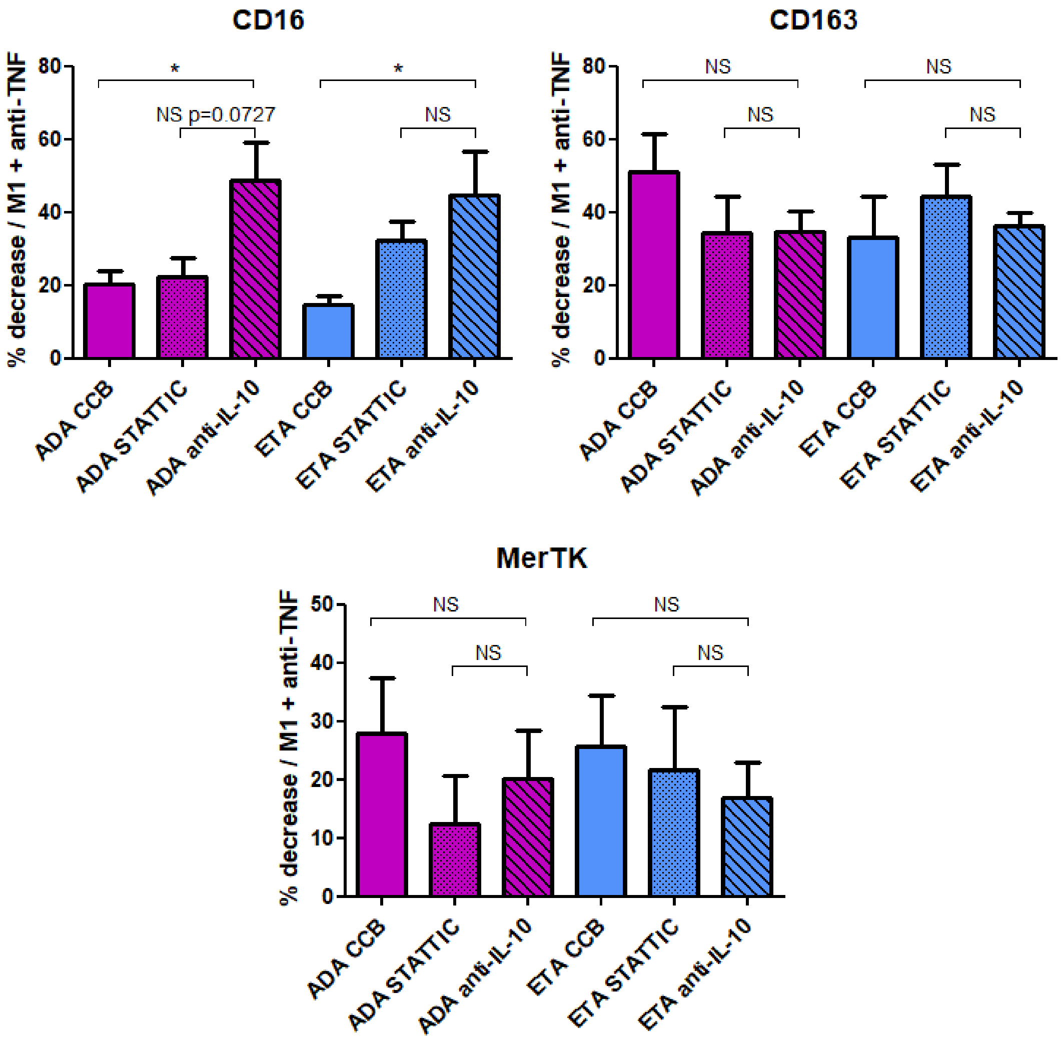

*ADA: adalimumab. Anti-IL-10: anti-interleukin 10 monoclonal antibody. CCB: cucurbitacin. ETA: etanercept. Inflammatory macrophages were activated for 24h in the presence of the indicated TNF inhibitor and the indicated inhibitor. Ability of the different inhibitors (cucurbitacin, STATTIC, anti-IL-10 monoclonal antibody) to affect the expression of membrane M(IL10) markers were compared by a Mann-Whitney test. *: 0.01≤p< 0.05*
